# The Technome - A Predictive Internal Calibration Approach for Quantitative Imaging Biomarker Research

**DOI:** 10.1038/s41598-019-57325-7

**Published:** 2020-01-24

**Authors:** Alexander Mühlberg, Alexander Katzmann, Volker Heinemann, Rainer Kärgel, Michael Wels, Oliver Taubmann, Félix Lades, Thomas Huber, Stefan Maurus, Julian Holch, Jean-Baptiste Faivre, Michael Sühling, Dominik Nörenberg, Martine Rémy-Jardin

**Affiliations:** 1 Department CT R&D Image Analytics, Siemens Healthineers, Forchheim, 91301 Germany; 2 Department of Radiology, University Hospital Großhadern, LMU, Munich, 81377 Germany; 3 Department of Medical Oncology, University Hospital Großhadern, LMU, Munich, 81377 Germany; 4 Comprehensive Cancer Center, University Hospital Großhadern, LMU, Munich, 81377 Germany; 5 Department of Thoracic Imaging, CHRU et Universite de Lille 2, Hospital Calmette, Lille, 59037 France; 6 Neuroinformatics and Cognitive Robotics Lab, University of Technology, Ilmenau, 98693 Germany

**Keywords:** Diagnostic markers, Cancer imaging, Biomedical engineering, Computational science

## Abstract

The goal of radiomics is to convert medical images into a minable data space by extraction of quantitative imaging features for clinically relevant analyses, e.g. survival time prediction of a patient. One problem of radiomics from computed tomography is the impact of technical variation such as reconstruction kernel variation within a study. Additionally, what is often neglected is the impact of inter-patient technical variation, resulting from patient characteristics, even when scan and reconstruction parameters are constant. In our approach, measurements within 3D regions-of-interests (ROI) are calibrated by further ROIs such as air, adipose tissue, liver, etc. that are used as control regions (CR). Our goal is to derive general rules for an automated internal calibration that enhance prediction, based on the analysed features and a set of CRs. We define qualification criteria motivated by status-quo radiomics stability analysis techniques to only collect information from the CRs which is relevant given a respective task. These criteria are used in an optimisation to automatically derive a suitable internal calibration for prediction tasks based on the CRs. Our calibration enhanced the performance for centrilobular emphysema prediction in a COPD study and prediction of patients’ one-year-survival in an oncological study.

## Introduction

Technical variation poses a problem for radiological quantification of biological structures – in particular in terms of morphological tissue characteristics. In computed tomography (CT), for instance, a change in scan protocol or reconstruction method may considerably vary observable texture in the acquired 3D image series and thus texture quantifying features. Therefore, status-quo radiology is mainly a qualitative effort in the sense that it relies on the radiologists’ experience who can usually integrate above-mentioned technical variation intuitively in their diagnosis. Correspondingly, most radiological tomographic reconstruction methods, also in magnetic resonance imaging (MRI), are optimised for qualitative, not quantitative, assessment. For the successful application of radiomics, high-throughput and high-content screening of standard-of-care medical images, or quantitative imaging biomarkers (QIB) research in general, though, this technical variation poses a much larger problem. Analysed features can be very sensitive to the impact of technical variation. That is, a feature can be strongly affected by a technical characteristic, e.g., by a slight streak artifact barely recognisable for the human reader. It is hence worth striving for appropriate techniques to reduce this negative impact of technical variation on extracted features and, moreover, to make subsequent statistical analysis resistant to such effects. In the scientific literature, mainly the impact of trivially reducible technical variation on features is analysed, e.g., by the Quantitative Imaging Biomarker Alliance (QIBA)^1^. In CT, this variation results from varying scan and reconstruction parameters or from acquisitions with entirely different scanners. For the most part, it is thus reducible by simply setting the associated parameters to constant values. Accordingly, the QIBA’s main goal is the optimisation and standardisation of scan protocols. This is often attempted by impact analysis of acquisition parameters or scanner types^2–7^.

Technical variation can however result from interaction between the image acquisition and individual patient characteristics, too, yielding both inter-patient noise and artifact variation. In CT, a corpulent patient with a larger cross-section will usually have a higher noise level within his body than a slimmer patient. This effect appears despite constant scan parameters as less quanta arrive at the detector. Another example is beam hardening that is stronger if the cross-section of the patient is larger: photons in the center then have a higher average energy than in the periphery. As this kind of technical variation can occur although extrinsic factors are kept constant, we call it the non-(trivially-)reducible technical variation. While regular technical variation results from variation of e.g. voxel spacing, reconstruction kernel or slice thickness, non-reducible technical variation is a result of variation of patient geometry and/or attenuation characteristics and expresses in noise or artifact variation, such as cupping artifacts. While scientists in the field of image acquisition and image reconstruction deal with such non-reducible technical variation decreasing its qualitative influence with advanced techniques (e.g. via tube current modulation^8^), it is not known which impact non-reducible technical variation has on derived quantitative features, e.g. radiomics, in relation to the examined biological or pathological variation.

The impact of technical variation on the evaluation of scientific questions was initially marked by Leek^9^. He has shown that for laboratory experiments the measurements were correlated with e.g. the date of the experiment. He therefore used the word surrogate as a feature indicative for the technical variation as the date or the humidity of the laboratory. He describes different data-driven procedures to identify such surrogates. Fortin expanded this concept and used the cerebrospinal fluid in MR images as a so-called control region (CR, for technical variation)^10^. A singular value decomposition (SVD) of the CR cohort variation is used to determine the main technical variation in the cohort. He then decomposed the voxel intensity distribution of the brain into an impact of the biological label and an impact of the main technical variation by a least-squares fit. Finally, the intensity distribution is adjusted for the fitted technical variation, which is why the method is called Removal of of Artificial Voxel Effect by Linear Regression (RAVEL). ComBAT^11^ is also an older technology from genomics that found attention recently as the method was capable of stabilising radiomics features for technical variation resulting from different imaging protocols^12^. In this method, a feature is decomposed into an additive and an multiplicative imaging protocol effect. The effects are then estimated by an empirical Bayes fit and removed from the feature by subtraction and division. These are statistical methods, i.e. a calibration is learned on the same data as it is applied and their main focus is the stabilisation of intensity distributions or features with regards to technical variation.

Surrogate features encoding technical variation also play a crucial role in our approach. Although technical variation can completely falsify a statistical analysis, in-house experiments show that machine learning classifiers such as a random forest can to a certain degree automatically learn and therefore compensate for technical variation when predicting a label if enough data and features are available. Our goal is therefore to automatically qualify and subsequently select surrogates from CRs to enhance prediction tasks associated with the actual target regions. We focus on predictive calibration with regards to a label in contrast to the statistical standardisation found in the literature. Whereas statistical standardisation learns the calibration on the same data as it is applied, we apply the calibration to unseen data.

## Method

**Figure 1:**
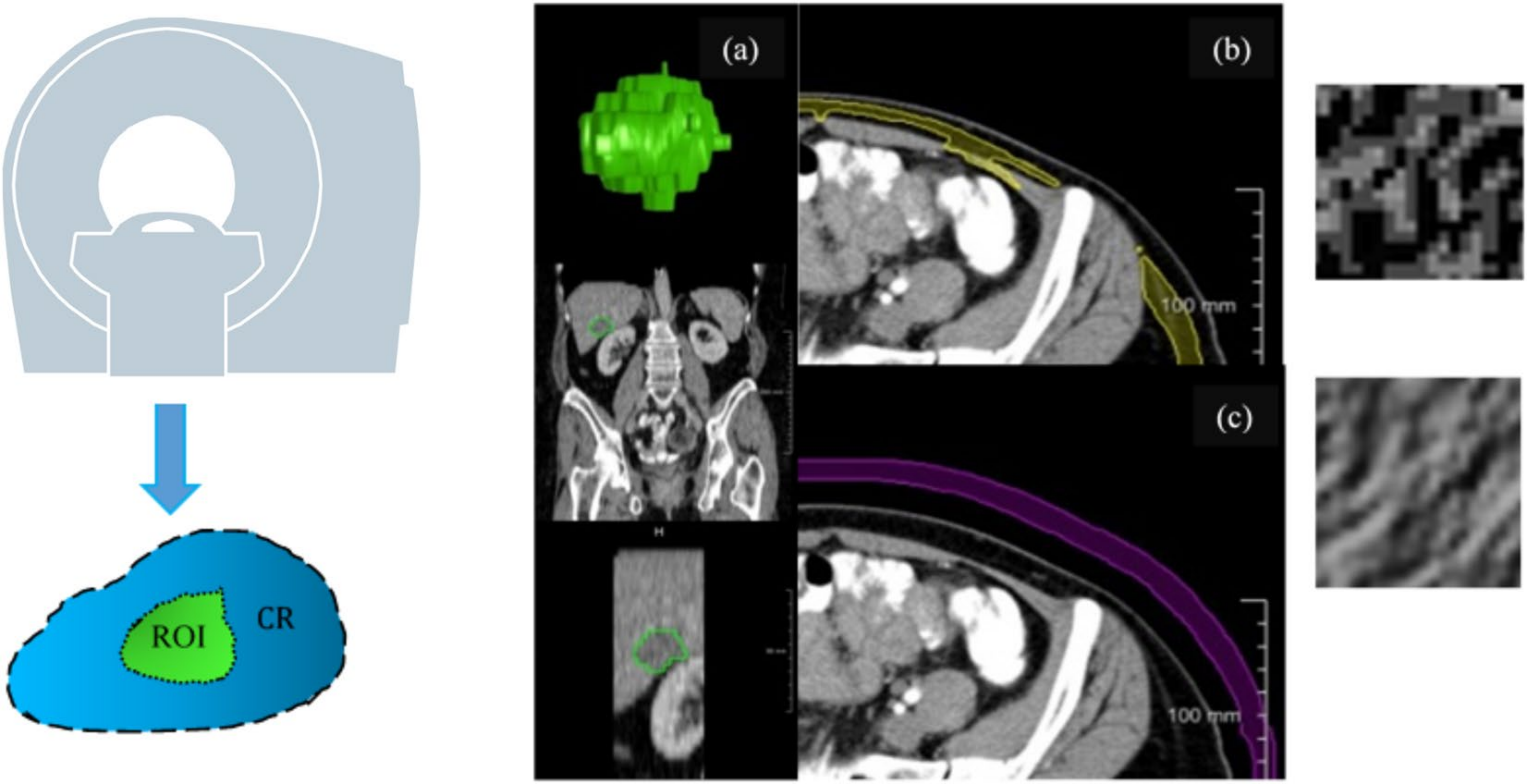
A tumour ROI (**a**) is internally calibrated by relating it to texture measurements in CRs such as adipose tissue (**b**) or air (**c**). It is assumed that *T*^*R*^ and *T*^*NR*^ are present in ROI and CRs.

As mentioned above we assume that the impact of both types of considered technical variation is not only present in a target region-of-interest (ROI), but also in a CR, a region inside the body which should ideally show only little inter-patient biological variation. The CR is thus assumed to store a patient-specific fingerprint of the inter-patient technical variation. Ideally, a CR should be close to a ROI in order to reduce the influence of spatially non-uniform noise and artifact distributions. Chambers of in-scan phantoms, which often serve as CRs, additionally placed next to the patient at scan time are particularly subject to this kind of inhomogeneity^13^. Besides, most clinical data is not acquired with in-scan phantoms. Our approach therefore merely relies on CRs that are naturally part of the imaging data to be processed. Regarding patient cohorts, the fingerprints of both regions, ROI and CR, induce an inter-patient correlation of biological and technical image information. For the sake of robustness, we extract surrogates from a multitude of CRs. We assume the entirety of surrogates over all CRs to contain the essential reducible and non-reducible technical information for relational quantification such as shown in Fig. 1. However, not all CRs and especially not all surrogates extracted from CRs are indeed suitable to represent technical variation. Thus they need to be qualified for this purpose, which we will describe later.

Reducible technical variation is termed *T*^*R*^ and non-(trivially-)reducible technical variation *T*^*NR*^. We call the entirety of features in a ROI a radiome ${({r}_{ij})}_{j\in R}:={({r}_{ij},\mathrm{..,}{r}_{iM})}^{\top }\in {{\mathbb{R}}}^{M}$ with patient index *i* and feature index *j* for a total of M extracted features. The relevant clinical annotation (or label) for patient i is termed *b*_*i*_. The general task of personalised medicine within imaging science is the generation of classification models which predict (machine learning) or explain (statistics) *b*_*i*_ by analysing ${({r}_{ij})}_{i\in P,j\in R}$ for the analysed patients *P*. This allows to design decision-support algorithms ${\hat{b}}_{i}={f}^{{\rm{clf}}}(({r}_{ij}{)}_{j\in R})$, where *R* is the set of used features. We define *S* as the set of used surrogates. Accordingly the surrogate space is termed *s*_*il*_ for *l* ∈ *S*.

### Explicit and implicit calibration in literature: *stabilisation* and *predictive* mode

To understand the dichotomy we will introduce in our calibration, we first have to explain the case separation of explicit and implicit calibration in the literature. It is a well-known problem that a calibration method that maximises the stability against technical variation may not be the method that enables optimal classification or prediction^14–16^. This is based on the fact that the focus of a calibration for the latter must lie on the discriminative part of the biological variation – and not the overall stability. We thus see two different operational modes of a calibration: *predictive* mode and *stabilisation* mode. The *stabilisation* mode yields the stabilised radiome only, while the *predictive* mode works with respect to the final prediction task.

The *stabilisation* mode is used to maximise the amount of information invariant to technical variation. RAVEL and ComBat use this mode as they explicitly remove the technical variation from the features. They first decompose the feature into technical and biological covariates. This happens by a linear fit. RAVEL fits the intensity distribution by the singular vectors of the CR intensities as determined by a SVD. The decomposition of RAVEL for the voxel $\overrightarrow{v}$ is,

1 $${I}_{i}(\overrightarrow{v})={\beta }_{0}+{\beta }_{b}{b}_{i}+\mathop{\sum }\limits_{k}^{N}\,{\beta }_{k}{{\rm{SVD}}}_{k}({I}_{i}(CR(\overrightarrow{v}\mathrm{)))}.$$

This fit yields the estimates ${\hat{\beta }}_{0}$, ${\hat{\beta }}_{b}$ and ${\hat{\beta }}_{k},k=\mathrm{1,}\,\ldots \,N$. The standardised intensity distribution then is,

2 $${I}_{i}^{\ast }(\overrightarrow{v})={I}_{i}(\overrightarrow{v})-({\hat{\beta }}_{k}+\mathop{\sum }\limits_{k}^{N}{\hat{\beta }}_{k}{{\rm{S}}{\rm{V}}{\rm{D}}}_{k}({I}_{i}(CR(\overrightarrow{v})))).$$

Translating the RAVEL principle from intensity distributions to features, we arrive at,

3 $${r}_{ij}^{\ast }={r}_{ij}-({\hat{\beta }}_{0}+\mathop{\sum }\limits_{k}^{N}\,{\hat{\beta }}_{k}{{\rm{SVD}}}_{k}(({s}_{il}{)}_{i\in P,l\in S})).$$

We term this approach RAVEL-like. Alternatively, the impact of covariates can linearly be removed after decorrelating them to select the most important ones: With ANCOVA, the feature can be decomposed into covariates^17^. Then a GLM fits the deviation of the feature from a fixed value by the deviation of covariates from their the average value within the cohort. By using surrogates as covariates this becomes $\Delta {s}_{il}={s}_{il}-{\bf{av}}{{\bf{g}}}_{i}(({s}_{il}{)}_{i\in P})$. In analogy to this covariate adjustment, the impact of qualified surrogates on the feature can be estimated by a linear fit,

4 $${r}_{ij}^{\ast }={r}_{ij}-({\hat{\beta }}_{0j}+\sum _{l\in S}\,{\hat{\beta }}_{lj}\Delta {s}_{il})$$

for surrogates ${s}_{il},l\in S$ and with the calibrated feature value ${r}_{ij}^{\ast }$ for *j* ∈ *R*. We see that the general form of the explicit calibration is,

5 $${r}_{ij}^{\ast }={r}_{ij}-{g}_{j}^{{\rm{reg}}}.$$

In RAVEL and ANCOVA, the function ${g}_{j}^{{\rm{reg}}}$ takes a linear form and is parametrised by singular vectors or covariates. The regression coefficients are determined by a decomposition of the feature and a linear fit. This approach however is per design extensible for a machine learning-based calibration procedure. The feature can of course also be fitted, or trained, by a machine learning model. For instance, ${g}_{j}^{reg}$ could be a random forest regression ${g}_{j}^{{\rm{RF}}}$ that predicts the deviation of the feature from a fixed value by the deviation of the surrogates from their mean. Subsequently, the predicted value can be subtracted from the feature value of the test data. This approach can be seen as a machine learning generalisation of the linear covariate adjustment. We will need this analogy later on for the validation of our method.

The *predictive* mode, on the other hand, assumes that not the whole image information needs to be stabilised for technical variation, but solely the image information that is needed for the classification task. Thus the invariance is optimised for the diagnostic relevant information. This approach is motivated by Leek^9^, who suggests to incorporate suitable surrogates for technical variation in the classification process. He also points out that it is important to identify suitable surrogates. In the field of laboratory experiments, surrogates such as ‘date of experiment’ or ‘laboratory personnel’ can be identified by a data-driven analysis. We, however, want to search CRs for suitable surrogates. We term the incorporation of these surrogates the *predictive* calibration mode. The classification process to predict the label *b*_*i*_ for patient *i* then becomes,

6 $${\hat{b}}_{i}={f}^{{\rm{clf}}}({({r}_{ij})}_{j\in R},{({s}_{il})}_{l\in S}),$$

where ${({s}_{il})}_{l\in S}$ are surrogate values for the patient *i*. The radiome is implicitly calibrated within the classification process by incorporating the qualified surrogates that have been shown to interact with the radiome technically and linearly.

Naturally, not all available surrogates should be used for explicit or implicit calibration, but only a qualified subset that is indeed suitable to calibrate the given features. Using all accessible surrogates without any qualification is termed a naive approach. Given enough data a classifier can to some degree compensate for technical variation even in a naive approach, however, we expect an increase in stabilisation and especially predictive performance when the surrogates are qualified for the given features which they shall explicitly or implicitly calibrate.

We term the refined classifier ${f}^{{\rm{clf}}}({({r}_{ij})}_{j\in R},({s}_{il}{)}_{l\in {S}^{q}})$ based on features and qualified surrogates *S*^*q*^ ‘technome *predictive* mode’. The set of regression models ${g}_{j}^{{\rm{reg}}},j\in R$ to explicitly calibrate a feature based on qualified surrogates for the respective feature is termed ‘technome *stabilisation* mode’. The technome can therefore have two modes, specialising on stabilisation or prediction. We now describe how the surrogates are qualified and the technome is constructed based on surrogate qualification for the respective mode.

## Technome Construction as a Model-Based Optimisation

**Figure 2:**
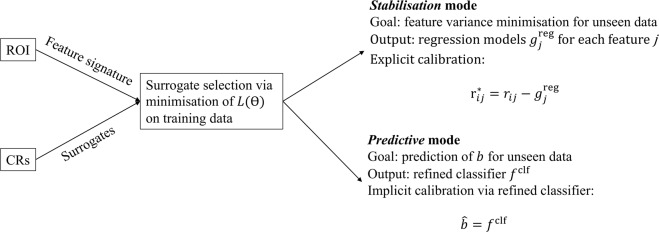
Overview of the method.

A diagram that gives an overview of this section is shown in Fig. 2. As explained above, used surrogates should not only be qualified for a suitable calibration but also enhance prediction. We denote a qualification score of a surrogate *l* ∈ *S* for the feature *j* ∈ *R* as *q*_*jl*_. Surrogates are qualified by *in vivo* assessment ${q}_{jl}^{{\rm{inVivo}}}$, *in silico* tests ${q}_{jl}^{{\rm{inSilico}}}$, phantom tests ${q}_{jl}^{{\rm{inVitro}}}$ and also for statistical reasons ${q}_{jl}^{{\rm{orthog}}}$. An exhaustive explanation and rationale of our qualification criteria can be found in the Supplementary Material (A.). Pseudo code of the method is found in Supplementary Material (B.). As the relevance of the different surrogate qualification criteria for the technome construction process is unknown, we have to introduce free weighting parameters $\Theta =\{{\theta }^{{\rm{inVivo}}},{\theta }^{{\rm{inSilico}}},{\theta }^{{\rm{inVitro}}},{\theta }^{{\rm{orthog}}}\}$ which we determine on training data. We therefore define a loss function that has to be minimised,

7 $$L(\Theta )={L}_{{\rm{train}}}(\Theta )+{L}_{{\rm{calib}}}(\Theta \mathrm{)}.$$

The performance for the respective task on the training data is introduced as *L*_train_. In *predictive* mode it should be a classification performance metric e.g. an Area-Under-Curve of the receiver-operating characteristic (AUC) for the given label, in *stabilisation* mode it should ideally be a metric that quantifies the stabilisation performance. For the technome *stabilisation* mode, the training loss may be introduced as the variance of the feature deviation from the cohort mean that can be explained by the surrogates’ deviation of the cohort mean. For the technome *predictive* mode, the training loss may be the predictive performance in a 10-fold cross-validation (CV) on the training data in case of a machine learning procedure. In case of a statistical classifier such as the logistic regression, we can simply use the discriminative AUC of the classifier on the training data.

We now calculate a qualification score *Q*_*jl*_ for each of the surrogates *l* ∈ *S* for each of the features *j* ∈ *R* of the radiome. We define the qualification score for surrogate *l* for the feature *j* and the parametrisation Θ as,

8 $${Q}_{jl}(\Theta )={\theta }^{{\rm{inVivo}}}{q}_{jl}^{{\rm{inVivo}}}+{\theta }^{{\rm{inSilico}}}{q}_{jl}^{{\rm{inSilico}}}+{\theta }^{{\rm{inVitro}}}{q}_{jl}^{{\rm{inVitro}}}+{\theta }^{{\rm{orthog}}}{q}_{jl}^{{\rm{orthog}}},j\in R,l\in S.$$

The set of qualified surrogates to calibrate the respective feature of the radiome is then defined as all surrogates with a qualification score higher than an arbitrary value of *Q*_*min*_ = 1.0. The weights Θ control which surrogates are selected for the internal calibration.

9 $${S}_{R,\Theta }^{q}=\{l\in S|{Q}_{jl}(\Theta ) > {Q}_{min},j\in R\mathrm{\}}.$$

For each feature *j* ∈ *R* of the radiome a subset of qualified surrogates is determined. By penalising large weights Θ, only surrogates with sufficiently high qualification values are selected by Eq. 9, thus semantically constraining the available image information used for calibration. The crucial point is now to enforce an internal calibration of the features by introducing the calibration loss as,

10 $${L}_{{\rm{calib}}}(\Theta )=-\,\alpha \,/(\sum _{\theta \in \Theta }\,\theta ),$$

in which a higher qualification of the surrogates translates to a lower value of *L*_calib_. The design parameter *α* is introduced to find a reasonable tradeoff between the used performance metric on the training data and the enforced internal calibration. A Bayesian optimisation process^18^ is used to determine the weights of the semantic constraints Θ that minimise the combined loss for training and qualification $L={L}_{{\rm{train}}}+{L}_{{\rm{calib}}}$ on the experimental data similar to systems-biology model building approaches^19^,

11 $$\Theta =\mathop{{\rm{argmin}}}\limits_{\Theta }({L}_{{\rm{train}}}+{L}_{{\rm{calib}}}).$$

The minimisation of *L* is achieved by Bayesian optimisation on training data. This determines the chosen weights Θ which in turn construct the technome.

We expect a higher predictive and stabilisation performance for unseen data when the surrogates have higher qualification and indeed internally calibrate the analysed radiome.

As a side effect, the technome can also be read out to discover new insights of feature stability in general (*stabilisation* mode) or technical impact on diagnosis (*predictive* mode). The qualification criteria scores *Q*_*jl*_ on the one hand help to determine suitable surrogates, on the other hand they also determine feature stability *in vivo*, *in vitro* and *in silico*. If many qualified surrogates can be found for a feature even for low values of Θ, the feature is non-robust. Finally, the weights Θ can help to explore whether either phantoms, simulations or associations in real data are most important to identify a suitable internal calibration that enhances predictive performance. The importance of different qualification criteria is to the best of our knowledge not yet analysed.

## Validation Strategy

**Figure 3:**
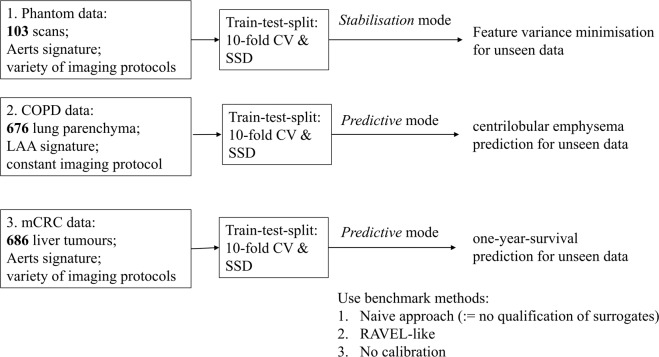
Overview of the validation strategy.

A diagram that gives an overview for this section is shown in Fig. 3. We employ a validation scheme similar to that of RAVEL^10^ that assesses if a calibration increases reproducibility of already shown associations between features and biological labels. For this purpose, we use two signatures that were often shown to be predictive for their use case: the Aerts signature^20^ for patient survival prediction in oncology and the low-attenuation area (LAA) signature consististing of LAA910 and LAA950 for emphysema assessment^21^. For the Bayesian optimisation needed to construct the technome, we integrated an established Python implementation^22^ in our pipeline. The acquisition function ‘upper confidence bound’, 8 initial seeds, 500 iterations and a Kappa of 5 as tradeoff between ‘exploration’ and ‘exploitation’ were chosen. An overview of all conducted experiments for *stabilisation* and *predictive* mode is shown in Table 1. Analyses were conducted with R packages (version 3.3.2, www.R-project.org) and scikit-learn^23^.

**Table 1:** Overview of all experiments.

Study type	Task	Data	*T*^*R*^	*T*^*NR*^	Surrogates	Features	Technome Mode
Phantom	Feature Stabilisation	103 CT Scan protocols for phantom	X	X	PyRadiomics features in CRs air, adipose tissue, liver, trachea, spleen, heart, aorta	Aerts signature in phantom ‘liver-tumours’	*Stabilisation*
COPD	CLE Prediction	676 lung parenchyma on CT		X		LAA signature in parenchyma	*Predictive*
	CLE SSD			X			*Predictive*
mCRC	1-ys Prediction	686 liver tumours on CT	X	X		Aerts signature in liver tumours	*Predictive*
			X	X			
	1-ys SSD		X	X			*Predictive*

Shown are the clinical field, task, used data, technical variation within the data, i.e. non-reducible *T*^*NR*^ or also reducible *T*^*R*^, used surrogates, features and technome mode. CLE: centrilobular emphysema, 1-ys: one-year-survival.

### Benchmark methods and train-test split

We compare the performance in technome *stabilisation* and *predictive* mode with the RAVEL-like approach and the naive approach, i.e. using all surrogates without any qualification criteria, as introduced above.

As a first test we compare the technome performance with a naive approach. As explained above a naive approach uses Eq. 5 or 6 respectively with all available surrogates without constrainining them. It is assessed whether the classifier or regressor can identify the relevant surrogates by themselves without additional qualification criteria. We test two cases for the stabilisation and prediction: a simple linear model *f* ^GLM^ or *g* ^GLM^ and a more complex, non-linear random forest classification, *f* ^RF^, or regression, *g*^RF^, model. To minimise redundant surrogates for the naive linear approach, surrogates are decorrelated via minimum redundancy maximum relevance (mRMR) algorithm^24,25^ and finally regularised by best subset feature selection according to the Akaike information criterion (AIC) to yield the set of used surrogates *S*.

The RAVEL-like approach uses the principal components of all available surrogates and uses formula 3 for the explicit stabilisation. For the predictive mode, the principal components are incorporated in the classifier. The principal components of the CR’s surrogates are determined via SVD. Principal components are collected until they explain 95% of the variance of the training data.

For technome *predictive* or *stabilisation* mode, we only assess linear approaches *f* ^GLM^ and *g*^GLM^ respectively. By concept, we explicitly enforce a linear calibration with the inVivo and inSilico qualification and expect a potential advantage to use a regularised, interpretable and statistically valid model on training data.

To test the stabilisation or prediction performance on unseen data, a 10-fold CV is used. Therefore, the data is split in training and test data, where the ratio of train to test data is 9:1. The training data is used to qualify the surrogates and optimise the weights Θ. This yields the technome *stabilisation* or *predictive* mode, i.e. the regression model for explicit feature calibration, *g*^GLM^, or the refined classifier for the medical label, *f* ^GLM^. The models are then applied for the unseen test data and the predictive or stabilisation performance is assessed. As very often in clinical scenarios only a small number of patients can be collected to detect the effect of a drug etc., the performance of the method in the small sample setting is assessed. We therefore choose the ratio of train to test data as 1:4 and term this scenario small sample detectibility setting (SSD) (Table 2). Both train-test split scenarios are summarised in Table 2.

**Table 2:** Validation schemes and the ratio of used data for training and test set.

Validation Scheme	Technome Computation and Training Set	Test Set
10-fold CV	9/10	1/10
SSD	1/5	4/5

### Phantom dataset - feature stabilisation

**Figure 4:**
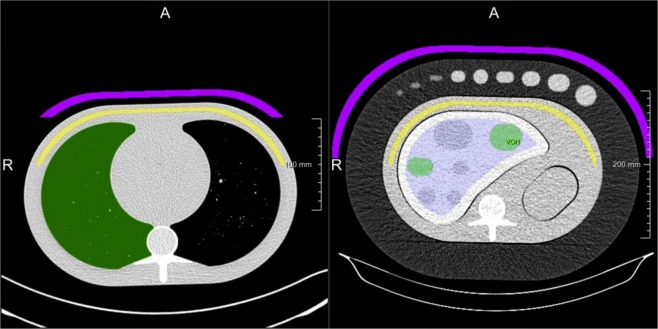
Lung phantom (left) and liver-lesion phantom (right) with CRs air (magenta) and ‘adipose tissue’ (yellow).

To validate the technome *stabilisation* mode, 103 CT images of a liver-lesion phantom^26^ acquired with a Siemens Somatom Zoom Scanner are analysed (Fig. 4, right). The images were acquired with a variety of scan and reconstruction parameters. Varied parameters are e.g. kvp, mAs, slice thickness, voxel size. The 3 texture features of the Aerts signature are extracted within a ‘tumour-like’ ROI and the 844 PyRadiomics^27^ features are used as surrogates *S* for the CRs air and ‘adipose tissue’. The ROIs are segmented by a semi-automated algorithm^28^.

The training loss is defined as variance *R*^2^ of feature *j* that is explained by *g*^GLM^ for the current qualified surrogate selection averaged over all analysed features ${L}_{{\rm{train}}}=-\,{\bf{av}}{{\bf{g}}}_{j}(({R}^{2}({g}_{j}^{{\rm{GLM}}}))$. For the calibration loss, only the qualification criterion ${q}_{jl}^{{\rm{inVivo}}}$ is used for phantom scans, as no biological induced variation needs to be excluded. Therefore ${L}_{{\rm{calib}}}=-\,\alpha {\theta }^{{\rm{inVivo}}}$ with *α* = 0.1 is enforced. The labels of the scan and reconstruction parameters are not used for the task.

The stabilisation performance on the test data is assessed by the variance reduction of the procedure i.e. the difference of feature variance before and after calibration ${{\rm{Perf}}}_{j}^{{\rm{stabil}}}=1-\frac{{\rm{var}}(({r}_{ij}^{\ast }{)}_{i\in P})}{{\rm{var}}(({r}_{ij}{)}_{i\in P})}$. The performance is then determined as the average performance for all features *j* ∈ *R*, ${{\rm{Perf}}}^{{\rm{stabil}}}={\bf{av}}{{\bf{g}}}_{j}({{\rm{Perf}}}_{j}^{{\rm{stabil}}})$.

### COPD dataset – centrilobular emphysema prediction

To validate the technome *predictive* mode ability to enhance centrilobular emphysema prediction in the presence of only non-reducible technical variation, 676 partly contrast-enhanced CT scans of 676 patients (age: 84.1 ± 14.8 y) each reconstructed with a soft (B36) and hard (B71) kernel, i.e. 1352 clinical CT images were acquired with a Siemens Somatom Force in Lille, France^29^. The cohort consists of patients with different symptoms of chronic obstructive lung disease (COPD). The study was approved by the local ethics commitee with waiver of the informed consent because CT examinations were part of routine clinical practice. Scan and reconstruction parameters are kept constant and only images reconstructed with kernel B71 are analysed, therefore no reducible technical variation exists. Additionally, tube current modulation techniques^8^ CareDose and CareKV were used to guarantee consistent image quality and further minimise non-reducible technical variation.

**Figure 5:**
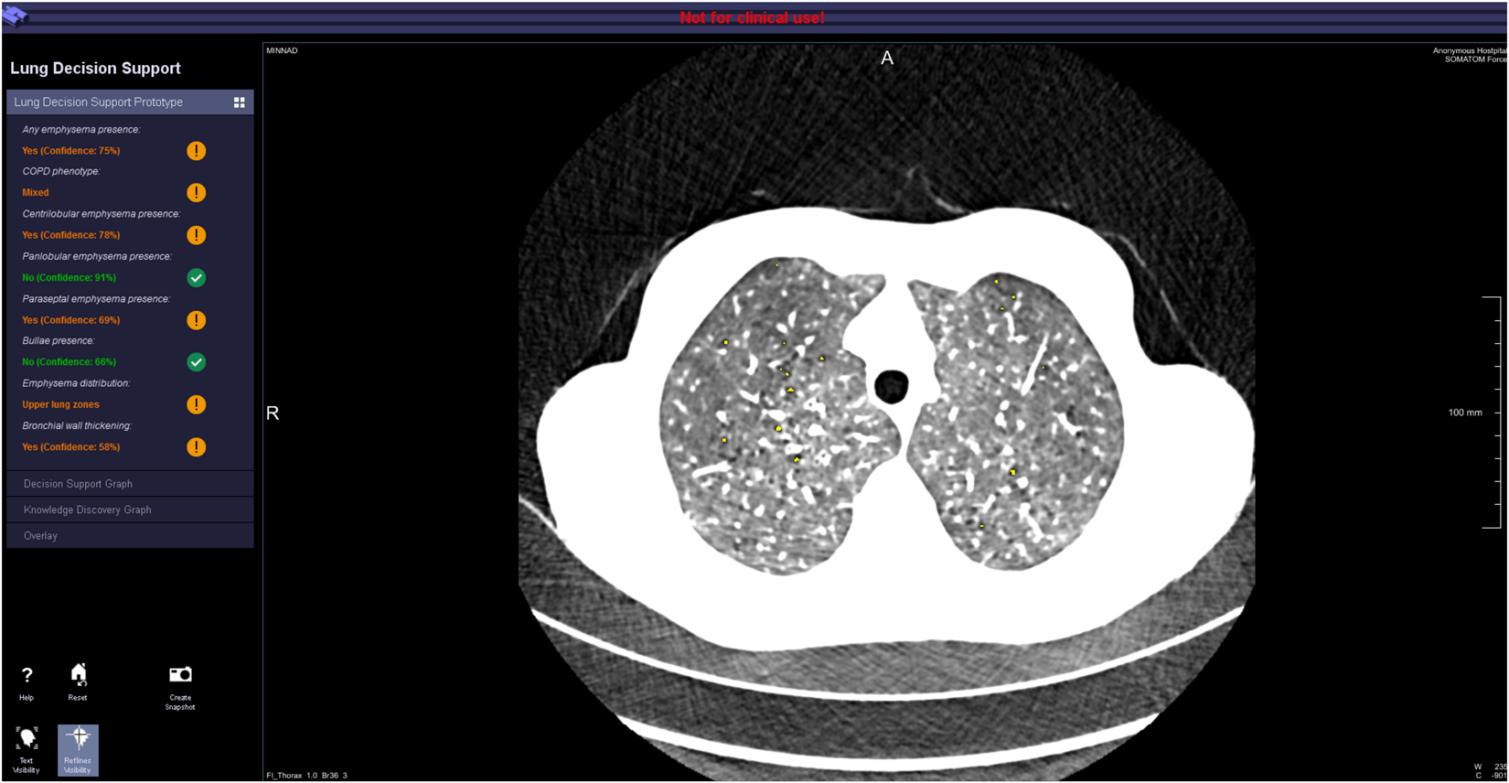
Prototype used for parenchyma analysis. An example of a COPD patient is shown.

The CRs air, trachea, adipose tissue, liver, heart, spleen and aorta segmented by an automated algorithm by Seifert *et al*.^30^. The lung parenchyma is segmented by an adaption of the deep learning segmentation of Yang *et al*.^31^ (Fig. 5). As features *R* we analyse the LAA signature. The LAA signature consists of two clinical standard features LAA910 and LAA950^21^. These features are commonly quantified to assess emphysema in clinical practice. Surrogates *S* are the 844 PyRadiomics^27^ features extracted in the CRs.

For the inSilico surrogate qualification ${q}_{jl}^{{\rm{inSilico}}}$, *in silico* variation was generated by superimposition of Gaussian, Rayleigh, Poisson and Gamma noise with 5 steps of monotonically increased noise each. For the inVitro surrogate qualification ${q}_{l}^{{\rm{inVitro}}}$, 16 CT images with varying scan and reconstruction parameters of the parenchyma in an anthropomorphic lung phantom^32^ (Fig. 4, left) were used.

The training loss is defined by the discriminative AUC of *f* ^GLM^ for the current selection of qualified surrogates on the training data *L*_Train_ = −AUC. The calibration loss is defined as ${L}_{{\rm{calib}}}=-\,\alpha /({\sum }_{\theta \in \Theta }\,\theta )$ with all qualification criteria and *α* = 0.2. The predictive performance is assessed as the AUC on the test data.

We compare the predictive performance of the technome for the hard kernel with the predictive performance of the LAA signature on the soft kernel. While a hard kernel is clinically considered inadequate for emphysema assessment due to larger inter-patient noise variation, we expect a predictive performance similar to the results for a soft kernel after calibration.

### mCRC dataset – one-year-survival prediction

**Figure 6:**
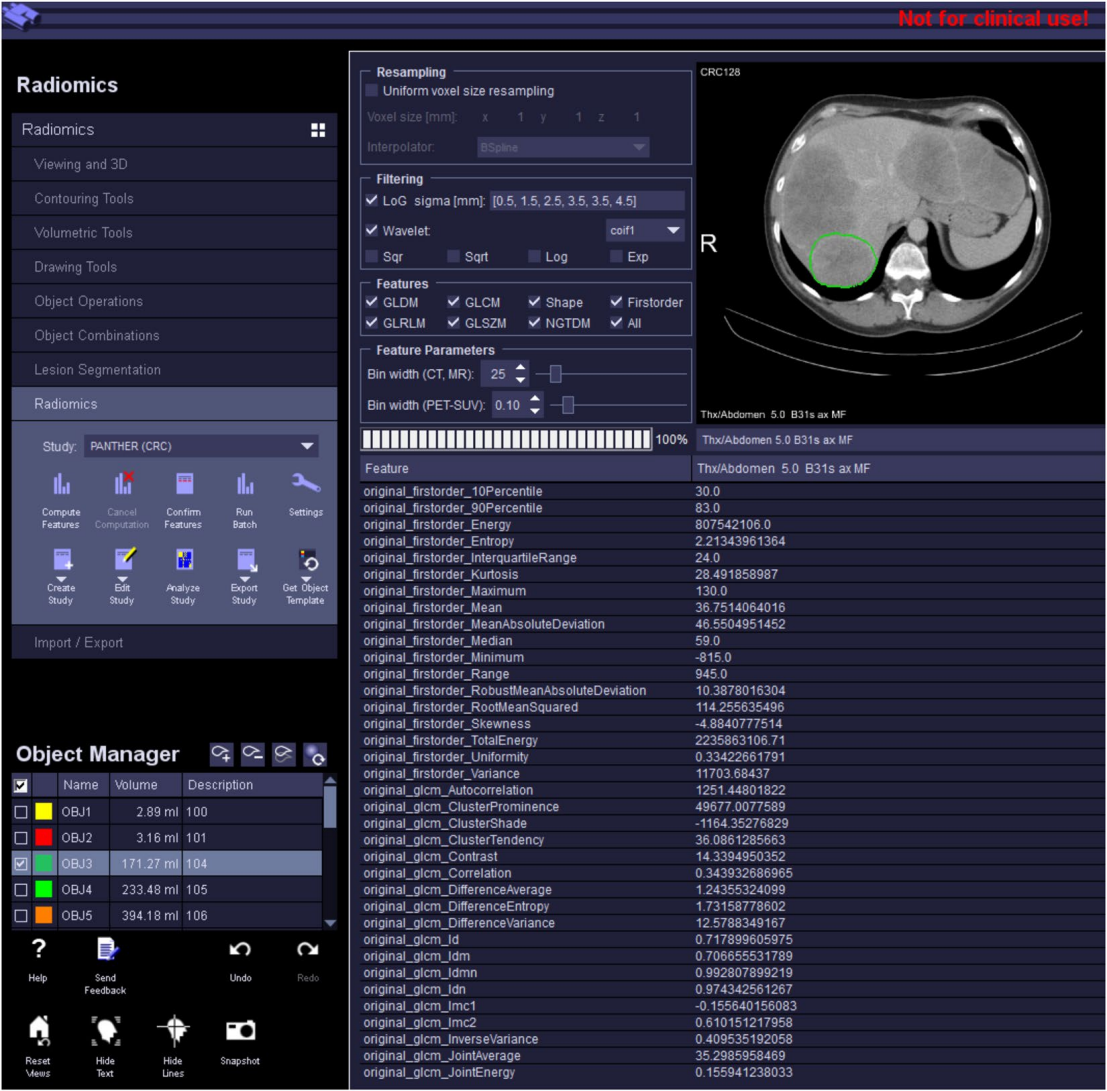
Radiomics prototype^34^ for PyRadiomics feature and surrogate extraction. An example of a mCRC patient is shown.

To validate the technome *predictive* mode ability to enhance one-year-survival prediction in the presence of non-reducible and also reducible technical variation, 118 contrast-enhanced CT scans of 75 patients (age: 61.9 ± 11.4 y) with 686 analysed liver tumours acquired with a variety of scanners from different vendours (GE, Philips, Siemens, Toshiba) in Munich, Germany, are analysed^33^. The cohort consists of metastatic colorectal cancer (mCRC) patients with liver metastases. The study was approved by the local ethics commitee with waiver of the informed consent because CT examinations were part of routine clinical practice. Scan and reconstruction parameters show large variation i.e. reducible and non-reducible technical variation is present. Tumours were segmented by a semi-automated segmentation algorithm^28^. The Aerts signature^20^ consisting of 4 features *R* is extracted from the tumour ROIs. Feature extraction and CR segmentation was conducted within a specialised radiomics framework^34^ (Fig. 6). Used surrogates *S* are the PyRadiomics features^27^ in the CRs air, trachea, adipose tissue, liver, heart, spleen and aorta again segmented by the algorithm of Seifert *et al*.^30^.

*In silico* variation for the calculation of ${q}_{jl}^{{\rm{inSilico}}}$ was generated by superimposition of Gaussian, Rayleigh, Poisson and Gamma noise with 5 increasing steps each. For *in vitro* variation, 103 CT images of an anthropomorphic liver-lesion phantom^26^ (Fig. 4, right) with a variety of scan and reconstruction parameters were used.

Training and test loss are defined in analogy to the COPD dataset. For each fold, the tumours of a patient were either in the training or the test data and a split was not allowed. Again, the predictive performance is assessed as the AUC on the test data.

For comparison purposes, all PyRadiomics features are extracted in the tumours and entered in a random forest classifier to predict one-year-survival. This random forest is optimised by hyperparameter tuning on the training data. With this step, we can assess, whether our calibration outperforms a hyperparameter tuned classifier using an abundance of features within the ROI. Finally, we compare the predictive performance of our technome classifer with an advanced deep learning method. For more details regarding the deep learning architecture we refer to Katzmann *et al*.^35^.

### Technome discovery – understand importance of qualification criteria and surrogates

Finally, it has to be analysed how qualification criteria are associated with the predictive performance. It is not clear whether an improvement of the predictive performance is really induced by an enforced internal calibration or simply by additional accessible biological information from the CRs. Therefore we have to assess whether qualification criteria indeed enforce an internal calibration. If this is the case, a higher qualification of surrogates should translate to a higher predictive performance. For the data presented above, each qualification criterion is inspected individually. For a first assessment, no loss is minimised as the goal is to analyse the association of qualification criteria with predictive performance without emphasising a certain subspace of the weight space Θ. For the inspection of individual qualification critera, *Q*_min_ is varied to generate 100 samples starting with 4 random seeds. Also combinations of qualification criteria are assessed for their association with predictive performance.

In a second step, it is assessed whether the minimisation of the loss *L* with *α* = 0.1 and only one qualification criterion, e.g. *L*_calib_ = −0.1/*θ*^inVivo^, yields acceptable predictive performance for each qualification criterion individually.

Finally, we read out the surrogates for calibration that yield the best performance for prediction of centrilobular emphysema and one-year-survival. Potentially, the integration of these surrogates could enhance clinical models using the LAA or Aerts signature.

## Results

### Phantom dataset – feature stabilisation

**Figure 7:**
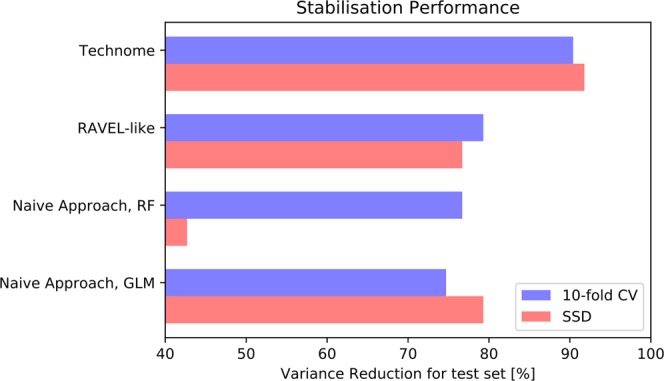
Stabilisation performance [% variance reduction] in 10-fold CV (blue) and SSD (red) in the phantom study.

The results are shown in Fig. 7. The technome *stabilisation* mode gives a stabilisation performance [% variance reduction on test set] in a 10-fold CV (SSD) of 90.4% (91.8%). The RAVEL-like calibration yields 79.3% (76.7%) and the naive approach via GLM 74.7% (79.3%) or via random forest 76.7% (42.7%).

Apparently, 21 training datasets of the SSD setting were not sufficient for a complex calibration as observed by the stabilisation performance of the random forest regression with all surrogates.

### COPD dataset – centrilobular emphysema prediction

**Figure 8:**
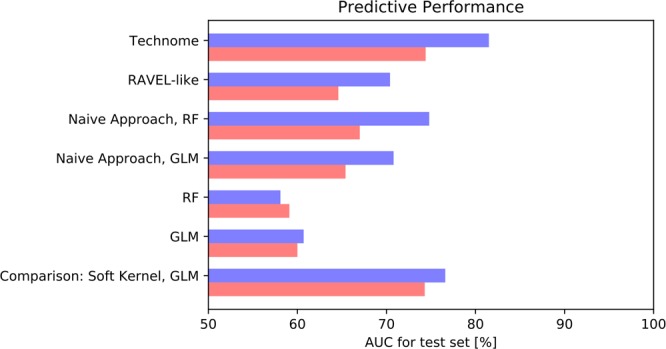
Predictive performance [% AUC] in 10-fold CV (blue) and SSD (red) for centrilobular emphysema prediction in the COPD study.

The results are shown in Fig. 8. The predictive performance [% AUC on test set] in 10-fold CV (SSD) improved to 81.5% (74.4%) with technome *predictive* mode in comparison to GLM with 60.7% (60.0%) or random forest with 58.1% (59.1%). The technome was also superior to a RAVEL-like calibration of 70.4% (64.6%) and the naive approach via GLM with AUC of 70.8% (65.4%) or via random forest with 74.8% (67.0%).

As a comparison, the predictive performance of LAA signature on the soft kernel B36, which is the clinical standard for emphysema classification, was 76.6% (74.3%), which is numerically inferior to the technome calibration for the hard kernel B71.

### mCRC dataset – one-year-survival prediction

**Figure 9:**
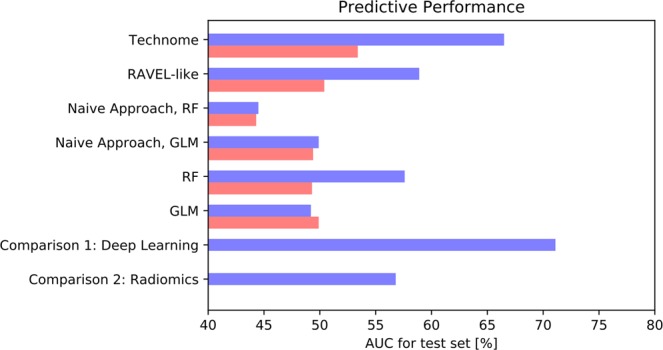
Predictive performance [% AUC] in 10-fold CV (blue) and SSD (red) for one-year-survival prediction in the mCRC study.

The results are shown in Fig. 9. The predictive performance [% AUC on test set] in 10-fold CV (SSD) improved to 66.5% (53.4%) with technome *predictive* mode in comparison to GLM with 49.2% (49.9%) or random forest with 57.6% (49.3%). The technome was also superior to a RAVEL-like calibration of 58.9% (50.4%) and the naive approach via GLM with AUC of 49.9% (49.2%) or random forest with 44.5% (43.3%). In the SSD scenario, no approach yielded an acceptable predictive performance. Apparently, the variation is too large to calibrate the signature with only few data.

For comparison, an advanced deep learning approach based on sparse autoencoder pre-training^35^ optimised for the same data achieved a performance of 71.1%. A radiomics approach with a random forest highly optimised by hyperparameter tuning using a nested CV and all PyRadiomics features calculated within the tumour ROIs achieves 56.8%.

### Technome discovery – understand importance of qualification criteria and surrogates

**Figure 10:**
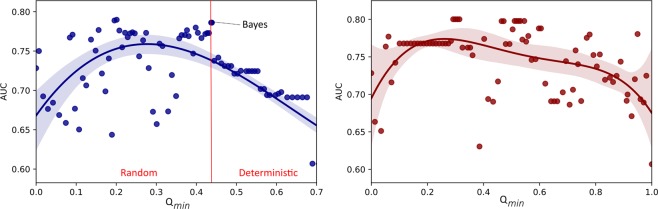
Association of the inSilico (left) and inVitro qualification (right) with the predictive performance for centrilobular emphysema. For high qualification the inSilico qualification criterion leads to a higher correlation of predictive quality with qualification, while the correlation of the predictive performance rapidly decreases for lower qualification. The inVitro qualification criterion when used alone, however, shows no obvious association with predictive performance. Only for inVivo and inSilico qualification criteria, the Bayesian optimisation constructed a technome calibration with a good predictive performance (‘Bayes’).

In a first step, the association of predictive performance with each qualification criterion individually was inspected. Apparently, only inSilico and inVivo qualification show a deterministic behaviour for high qualifications. This is shown exemplarily for centrilobular emphysema and the inSilico qualification in Fig. 10 on the left. The inVitro and orthogonality qualification used alone, however, are not associated with predictive performance. This is shown exemplarily for the inVitro qualification on the right in Fig. 10. When the loss *L* is minimised, the tradeoff between training loss and calibration loss used in the Bayesian optimisation detects a point of good predictive performance for the inVivo and inSilico qualification within the deterministic area (‘Bayes’ in Fig. 10).

**Figure 11:**
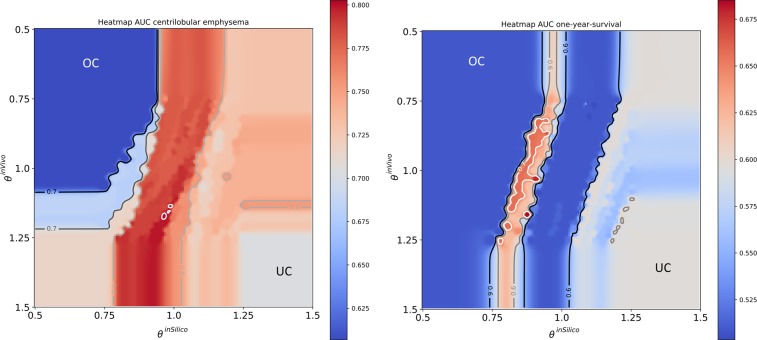
Association of combined inVivo & inSilico qualification weights with the predictive performance [%AUC on test data] for centrilobular emphysema (left) and one-year-survival (right). Overconstrained (OC), i.e. no calibration, and underconstrained (UC) regions, i.e. also surrogates with low qualification are used, show lower predictive performance than models, where the analysed features were implicitly calibrated by highly qualified surrogates. Lower weights Θ translate to a higher qualification.

Therefore, we inspect the association of combined inVivo and inSilico qualification weights with predictive performance in Fig. 11. For the inspection of combined inVivo and inSilico weights, $\Theta =\{{\theta }^{{\rm{inVivo}}},{\theta }^{{\rm{inSilico}}}\}$, 500 random samples are drawn uniformly with fixed *Q*_min_ = 1.0. As can be seen in Fig. 11 the predictive performance is lower when all or many surrogates are used (underconstrained, UC) or no surrogate is qualified enough and thus no calibration is used (overconstrained, OC). Please note that surrogates with the highest qualification are found in direct border to the OC region and lower weights translate to a higher qualification of the surrogates as a consequence of Eq. 9. When only non-reducible technical variation is present, i.e. scan and reconstruction parameters are constant within the study, the inSilico qualification appears to be more important for the predictive performance as observed for centrilobular emphysema prediction in comparison to one-year-survival prediction, where also reducible technical variation is present. A good predictive performance for one-year-survival was achieved when inSilico and inVivo qualification were combined. A high qualification according to both criteria translates to a high predictive performance, while a larger weight for the inSilico qualification guarantees a higher predictive performance for emphysema presence.

In both studies a high qualification of surrogates according to the defined qualification criteria is associated with enhanced predictive performance. Though their individual inspection did not show any association with predictive performance, the additional integration of inVitro and orthogonality qualification still increased predictive performance numerically in comparison to the combined inVivo and inSilico qualification.

The surrogates that guaranteed the highest AUC on test data are shown in Table 3. The LAA signature is calibrated best by texture metrics within a cylindrical CR in the trachea, while the Aerts signature is calibrated by a combination of texture metrics in liver and heart. This could potentially be due to applied contrast injection variation that has to be calibrated.

**Table 3:** Technome discovery.

Signature	CR	Qualified surrogates
**Aerts signature**		
Grey Level NonUniformity	Heart	WL_LHH_GLSZM_LargeAreaEmphasis
	Liver	GLSZM_SizeZoneNonUniformity, firstorder_Entropy
	Trachea	WL_LHL_GLRLM_GrayLevelNonUniformity, WL_HHL_GLSZM_LargeAreaHighGrayLevelEmphasis, WL_HLL_firstorder_Uniformity
	Adipose Tissue	GLCM_Idmn
Statistics Energy	Liver	GLRLM_HighGrayLevelRunEmphasis, WL_LLL_firstorder_Entropy
	Heart	firstorder_Entropy
	Air	WL_HLH_firstorder_10Percentile
Grey Level NonUniformity HLH	Liver	GLSZM_SizeZoneNonUniformity, GLCM_Imc2
	Heart	WL_HLH_GLSZM_ZoneEntropy, WL_LHH_GLSZM_LargeAreaEmphasis
**LAA signature**		
LAA950	Trachea	GLDM_GrayLevelVariance, LS_0_5_mm_3D_GLDM_DependenceNonUniformityNormalized, GLDM_DependenceNonUniformity, GLDM_LargeDependenceEmphasis
	Air	GLDM_LowGrayLevelEmphasis
	Spleen	LS_2_5_mm_3D_GLDM_DependenceVariance, GLDM_DependenceVariance
	Aorta	LS_3_5_mm_3D_GLDM_DependenceNonUniformityNormalized
LAA910	Trachea	LS_3_5_mm_3D_NGTDM_Complexity, LS_0_5_mm_3D_GLDM_LargeDependenceEmphasis
	Heart	LS_3_5_mm_3D_GLDM_GrayLevelNonUniformity, LS_0_5_mm_3D_GLDM_DependenceNonUniformity, LS_0_5_mm_3D_GLDM_LargeDependenceEmphasis

The qualified surrogates that led to the best predictive performance for one-year-survival (top) and centrilobular emphysema presence (bottom) are shown. CRs are ordered according to their explained variance of the respective feature.The sphericity of the Aerts was robust to technical variation and is thus omitted. Abbreviations for filters - LS: logSigma, WL: Wavelet. Further explanations about PyRadiomics classes’ nomenclature such as ‘GLSZM’ can be found in the original publication^27^.

## Discussion

On CT images, all measurements should be seen as relative – a notion that is already accepted for bone mineral density measurements that are therefore calibrated by in-scan phantoms as seen, e.g., in the work by Kalender^36^. For bone mineral density, a calibration relates the measurements in a ROI to quantifications in the in-scan phantom. A univariate feature analysis without calibration thus appears dubious in most other cases as well. However, a machine learning algorithm can to some degree automatically calibrate for technical variation when predicting a label. We also observed this effect in our COPD study, where a naive approach that incorporated all available surrogates from non-lung CRs improved the predictive performance from 60.7% to 74.8%. To assist the classifier, we integrated an internal calibration based on surrogate qualification criteria in the training process of the classifier to enhance predictive performance. A feature signature indicative for the medical label is enhanced by additional incorporation of qualified surrogates. The qualification criteria for the surrogates stem from other sources of information usually used in radiomics feature stability analyses, such as phantom measurements, simulations but also best practices in statistics. For instance, our statistical qualification criterion enforces valid covariates to be used, as for a proper statistical assessment no interaction effect between the examined biological variable and the used covariate should occur. The introduced inSilico qualification criterion rewards the use of surrogates, when their association with the analysed feature *in vivo* can also be reidentified in simulation studies, which makes it highly unlikely that the association beween feature and surrogate is induced biologically. As in our case the technome classification uses a simple logistic regression model, the results can also be used for interpretable statistical assessment. The surrogates that are used within the model are appropriate for this task, as they have been shown to be suitable covariates by *in vivo*, *in vitro*, *in silico*, and orthogonality tests.

Our approach based on regularised models in which established imaging biomarker signatures are internally calibrated yielded better predictive performance than the signatures without calibration. Our models also outperformed conventional machine learning models with all available features and surrogates. This can be interpreted as an enhanced reproducibility of imaging biomarkers. Only an advanced deep learning approach^35^ that employs a sparse autoencoder for dimensionality reduction performed better to predict one-year-survival. However, deep learning lacks the interpretability and probably also the reproducibility of our approach. This is especially important as the lack of interpretability and especially reproducibility^37^ is already recognised as a large problem, not only for clinical studies, where a significant result of a potential biomarker associated with a treatment can often not be reproduced in similar studies. While the Imaging Biomarker Standardisation Initiative (IBSI)^38^ adresses this problem by introducing unified feature definitions, the minimisation of technical variation remains a pressing issue.

First, the technome improved centrilobular emphysema prediction with a hard kernel and using the LAA signature from an AUC of 60.7% to 81.5%. Possibly, this could be of clinical value as a hard kernel was previously considered inadequate for emphysema assessment due to the inter-patient noise variation. The prediction of emphysema based on LAA features only with a soft kernel even showed a slightly worse performance of 77%. Second, by calibration, the Aerts signature to predict one-year-survival reproduced its expected predictive performance with an AUC of 66.5%. In the original study^20^ this signature yielded a concordance index, which is a generalisation of an AUC, of 66–69% for unseen data. The prediction of one-year-survival based on the calibrated Aerts signature was superior in comparison to no calibration (49.2% via GLM and 57.6% via random forest), an optimised radiomics approach that uses all PyRadiomics features within the tumours and a nested cross-validation process (56.6%), and even comparable to the most advanced ‘black-box’ deep learning approaches (71.1%). Using a naive approach and entering the signature and all available surrogates in a random forest classifier led to an predictive performance that was even worse compared to no calibration (44.5% vs. 57.6%).

Despite not being our main focus, a second field of research was the explicit feature stabilisation in a phantom study. Although reducible technical variation can be read out via imaging protocol parameters, its information is sparse. For example the kernel is only a factor with no continuous parametrisation. This is also a limitation of the ComBat method, where for each imaging protocol a minimum number of cases must be available^12^. Therefore, it is not ensured that enough data for the respective imaging protocol can be collected. Also, a learned stabilisation, such as the deep learning approach by Jin^39^, does of course not work for unseen scan and reconstruction parameters. In our phantom study, the calibration is applied on unseen scan and reconstruction parameters and therefore not even one sample for each imaging protocol can be used to learn the calibration. ComBat does by design not work in this scenario. The technome parametrises reducible and non-reducible technical variation in a unified manner via qualified surrogates, while the information which scan and reconstruction parameters are employed is not used. We compared the technome stabilisation to an adaption of the well-known RAVEL method^10^, which is an advanced and well-performing MRI technique, that uses the singular vectors of variation in CRs to stabilise intensity distribution in MRI scans of the brain. The variance reduction performance of our method (90.4%) was numerically superior to RAVEL-like approach (76.3%) in the phantom study where object size, scan and reconstruction parameters were varied. This may be due to the fact that RAVEL uses the principal components of the CRs. Accordingly, each feature is calibrated by a selection from the same principal components of variation that are determined by a SVD of the CR’s surrogates. The voxel value, in our case the feature value, is then linearly adjusted for those principal components of variation. From our phantom experiments however, it appears that each feature needs a more specific calibration that can profit from a surrogate qualification. The principal components may not always be appropriate, a feature that is affected by a cupping artifact may not be properly calibrated if the cupping artifact is not expressed in the principal components. Again, the combination of a qualification criterion and a regularised model performed better than a naive approach using all surrogates (90.4% vs. 76.7% variance reduction).

The technome discovery indicates that technical variation can have an impact on features, even when scan and reconstruction parameters are kept constant within a study. One finding of our experiments is that the best predictive performance was achieved when surrogates are used that are qualified by the inVivo and the inSilico qualification metric. The inVivo qualification rewards when surrogates are used that are correlated with the feature. The inSilico qualification however analyses if the found correlation between feature and surrogate can be reproduced in simulations of technical variation. For constant scan and reconstruction parameters, simulations even appear necessary to qualify appropriate surrogates. The finding that a high qualification of the surrogates translates to a higher predictive performance explains the good prediction results that were achieved by introducing the calibration loss in both clinical studies. As the best predictive performance was observed for highly qualified surrogates, it is very probable that the performance enhancement is due to the enforced internal calibration procedure and not due to integration of further biological information from the CRs. As a side effect we tested many different organs for imaging biomarkers, as the two studies were an exhaustive multi-organ analysis for COPD and mCRC. However, the addition of all available surrogates from all organs without qualification showed numerically inferior performance to the technome prediction. In the mCRC study, the integration even led to deterioration of predictive performance. We expect this to be a result of overfitting. Furthermore, the chosen CRs for calibration were plausible. The LAA signature measured in the parenchyma was internally calibrated by the representation of air within a cylinder in the trachea. For the mCRC data with contrast injection the technome used texture metrics within liver and heart. Interestingly, the technome chose the heart CR for calibration of contrast injection and not the aorta. This may be due to a more constant accumulation of contrast within the blood pool of the heart.

Our method has several limitations. The main limitation is the dichotomy of *predictive* and *stabilisation* mode. This case separation should ideally not be needed, as a perfect explicit calibration should also enhance predictive performance without knowledge of the medical label. Second, we did not calculate Kaplan-Meier curves for survival prediction, which would allow a better comparison of the Aerts signature’s performance with the original study. An integration of the *predictive* mode within survival analysis would have needed further methodological effort. Third, we used only simple simulations and qualified them with the similarity argument of the inSilico qualification criterion. However, we expect an increase in predictive and stabilisation performance when more complex simulations are applicable, which was not yet possible due to the large computation time for each case. Fourth, our method in its current form is very computation-intensive and time-consuming. Especially the inSilico qualification needs to synthetically manipulate the images to collect associations between features and surrogates. Fifth, although we qualify surrogates for a feature by a variety of criteria, it is still possible that the non-uniform noise and artifact distribution in CT images can lead to the selection of suboptimal surrogates. The noise texture in the ROI can still significantly differ from the technically-induced texture that is found in the CR. Finally, of course, more data and clinical prediction tasks, e.g. for COPD^21^, liver^40^, or muscle diseases^41^, are needed for further validation of the method. As a further proof of concept, the predictive performance of established imaging biomarkers should be reproduced in studies with larger technical variation.

In conclusion, we present a novel method that integrates qualification criteria for surrogates in the training of a classification or regression method leading to a predictive internal calibration. The method improved feature stabilisation in a phantom study, prediction of centrilobular emphysema in a COPD study and one-year-survival in mCRC study. The analysed studies indicate that the identification of surrogate qualification criteria and their integration in the training process of a prediction model is a promising field of research.

In the future, we plan to combine the surrogate qualification with ComBat and deep learning. For the deep learning variant, the calibration loss could be included as a regularisation term that controls the incorporation of CRs in the classification process. For ComBat, the substitution of the imaging protocol by qualified surrogates seems to be an interesting option.

## Supplementary information

Supplementary Material.

## Data Availability

The relevant data supporting the findings are available within the article. The clinical study data are available from the corresponding author A.M. upon request.
