# Publisher Correction: The Technome - A Predictive Internal Calibration Approach for Quantitative Imaging Biomarker Research

**DOI:** 10.1038/s41598-020-68534-w

**Published:** 2020-07-07

**Authors:** Alexander Mühlberg, Alexander Katzmann, Volker Heinemann, Rainer Kärgel, Michael Wels, Oliver Taubmann, Félix Lades, Thomas Huber, Stefan Maurus, Julian Holch, Jean-Baptiste Faivre, Michael Sühling, Dominik Nörenberg, Martine Rémy-Jardin

**Affiliations:** 1 Department CT R&D Image Analytics, Siemens Healthineers, 91301 Forchheim, Germany; 2 Department of Radiology, University Hospital Großhadern, LMU, 81377 Munich, Germany; 3 Department of Medical Oncology, University Hospital Großhadern, LMU, 81377 Munich, Germany; 4 Comprehensive Cancer Center, University Hospital Großhadern, LMU, 81377 Munich, Germany; 5 Department of Thoracic Imaging, CHRU Et Universite de Lille 2, Hospital Calmette, 59037 Lille, France; 6 Neuroinformatics and Cognitive Robotics Lab, University of Technology, 98693 Ilmenau, Germany

Correction to:* Scientific Reports* 10.1038/s41598-019-57325-7, published online 24 January 2020

This Article contains an error introduced by the journal during the typesetting of Equation 2.

$$ I_{i}^{*} \left( {\vec{v}} \right) = I_{i} \left( {\vec{v}} \right) - \left( {\hat{\beta }_{k} + \mathop \sum \limits_{k}^{N} \hat{\beta }_{k} {\text{SVD}}_{k} \left( {I_{i} \left( {CR\left( {\vec{v}} \right)} \right)} \right)} \right). $$

should read:

$$ I_{i}^{*} \left( {\vec{v}} \right) = I_{i} \left( {\vec{v}} \right) - \left( {\hat{\beta }_{0} + \mathop \sum \limits_{k}^{N} \hat{\beta }_{k} {\text{SVD}}_{k} \left( {I_{i} \left( {CR\left( {\vec{v}} \right)} \right)} \right)} \right). $$

