# Supplementary material for: The Technome - A Predictive Internal Calibration Approach for Quantitative Imaging Biomarker Research
Source: Sci Rep. 2020 Jan 24;10:1103. doi: 10.1038/s41598-019-57325-7 (PMC6981189; doi:10.1038/s41598-019-57325-7)
Supplement: Supplementary file 1 — Supplementary Material. [file 41598_2019_57325_MOESM1_ESM.pdf]

# Supplementary Material: The Technome - A Predictive Internal Calibration Approach for Quantitative Imaging Biomarker Research

Authors: Alexander Mühlberg, Alexander Katzmann, Volker Heinemann, Rainer Kärgel, Michael Wels, Oliver Taubmann, Félix Lades, Thomas Huber, Stefan Maurus, Julian Holch, Jean-Baptiste Faivre, Michael Sühling, Dominik Nörenberg, Martine Rémy-Jardin

## A. Qualification Criteria: SALSA

SALSA (Similar Additive Linear Surrogate Association) is the acronym of the used set of surrogate qualification criteria. Most qualification criteria are based on the decomposition of the variation of each feature $j$ of the radiome in in vivo cohorts into technical variation and biological variation. The technical variation is parametrised by the surrogate $l$ while the technical variation is parametrised by the clinical label $b_i$ for patient $i \in P$,

$$r_{ijl} = r_{0j} + \beta_{b,jl} b_i + \beta_{s,jl} s_{il}, j \in R, l \in S. \quad (1)$$

Applying a GLM fit to this decomposition yields estimates of the regression coefficients (or slopes) $\hat{\beta}_{s,jl}$ and $\hat{\beta}_{b,jl}$. A used surrogate $l$ should ideally be an image feature that is prone to the impact of technical variation and not to biological variation. The purpose of the surrogate $l$ is to exclusively describe the impact of technical variation on the feature $j$. Only few surrogates that can be extracted in CRs qualify for this task. Therefore, qualification criteria are needed to use only appropriate surrogates. SALSA can be divided in a collection part, where suitable surrogates are proposed in a high-content and high-throughput screening of in vivo image data, and a exclusion part, where information from other sources is used to constrain the number of surrogates to a few semantically meaningful ones, which most probably interact with the radiome on a technical pathway.

### SALSA Collection Part

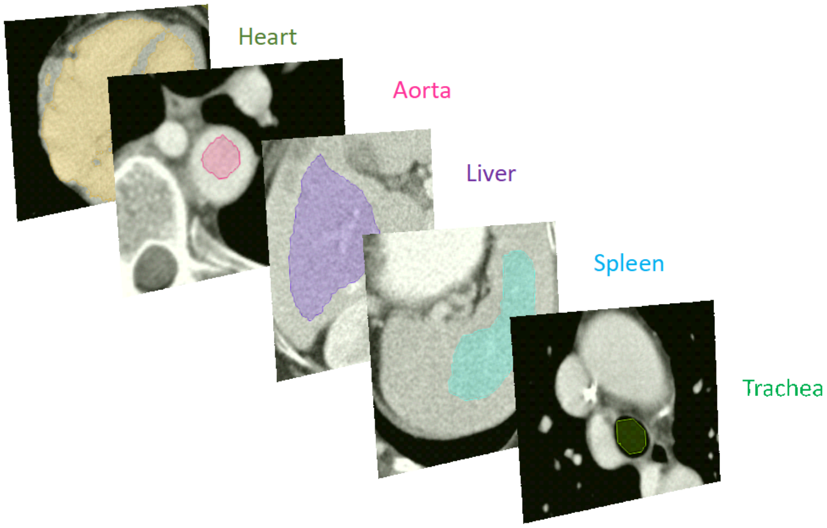


**Figure 1.** Automatically segmented control regions heart, aorta, liver, spleen and trachea.

As explained above, in the collection part, surrogates are extracted within CRs and a first qualification criterion is introduced to collect only relevant surrogates. To get CRs, stripes in air and adipose tissue are segmented with a simple constrained region growing-based on attenuation and the distance to the object boundary. We use the algorithm of Seifert[1] to automatically segment liver, spleen, aorta, trachea and heart (Fig. 1). The specificity of the organ segmentation is increased by simple morphological operations, such as erosion and opening, to obtain pure tissue representatives. In the CRs an exhaustive extraction of potentially qualifying surrogates for technical variation is performed.

**InVivo Qualification – Association of image information in vivo.** The first qualification criterion assumes that an observable inter-patient association between surrogate $l$ in a CR and a feature $j$ in a ROI is always a hint for technical variation, i.e. high correlations between a feature extracted in a ROI (e.g. a tumour region) and surrogates extracted in CRs (e.g. air and adipose tissue) are probably not induced biologically but technically[2,3] (Fig. 2). For example, adipose tissue is assumed to show

only little inter-patient biological variation[4]. A surrogate $l$ that is correlated with the examined feature $j$ is thus preferred for calibration. The Pearson correlation coefficient $R_{\text{Pearson}}$ between each feature of the radiome and each possible surrogate is calculated to quantify this association. The formulated in vivo qualfication for surrogate $l$ given a feature $j$ thus gives values in the interval [0;1],

$$q_{jl}^{\text{inVivo}} = |R_{\text{Pearson}}((r_{ij})_{i\in P}, (s_{il})_{i\in P})|. \quad (2)$$

For technical feasibility, i.e. an acceptable computation time, only surrogates with $R_{\text{Pearson}} > 0.15$ are passed to the exclusion part of the SALSA pipeline.

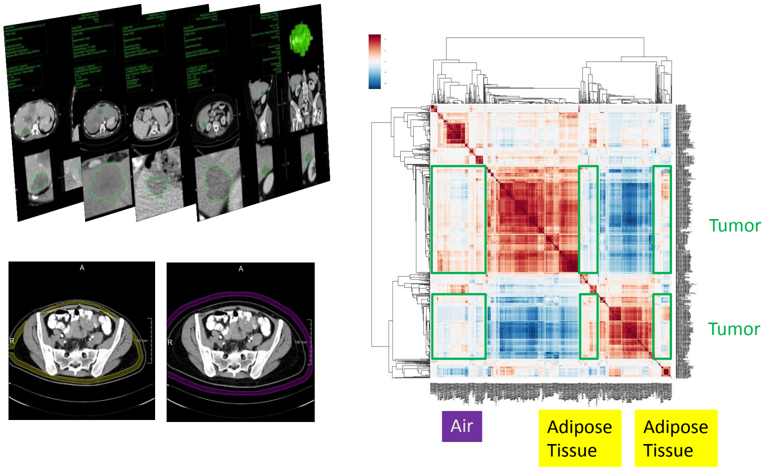


**Figure 2.** InVivo qualification. The inVivo qualification criterion quantifies the Pearson correlation (positive: red and negative: blue) between radiome (top left) and CR's surrogates (bottom left) in real in vivo study cohorts. Exemplary, the Pearson correlation heatmap with dendrogram of the radiome with surrogates in air and adipose tissue is shown (right). The examined associations are highlighted (green).

## SALSA Exclusion Part

Those associations found in high-throughput and high-content feature analysis of patient cohorts are subsequently analysed in more detail, as for many surrogates in CRs a biological association with the features in the ROI is also possible. Therefore the probability of a biological association mistakenly considered a technical association has to be minimized. We incorporate information from other sources, such as simulations and phantom studies as further qualification criteria to exclude associations of the collection part that are only coincidental or induced directly by the patient biology.

**InVitro Qualification: Surrogate examination in vitro.** To minimise biologically induced variation of surrogates for calibration, we use regions of anthropomorphic phantoms as CRs and quantify the relative change of surrogates for varying image quality due to different scan and reconstruction parameters as well as object sizes of the phantom. Surrogates are preferred that show strong response to technical variation in comparison to biological variation of the CR. The latter can be deduced if surrogates quantified in CRs are affected by a disease or physiological changes. We account for the impact of such changes by dividing of the standard deviation $\sigma^{\text{inVitro}}((s_{il})_{i\in\text{inVitro}})$ of the surrogate in the phantom study by its standard deviation $\sigma^{\text{inVivo}}((s_{il})_{i\in\text{inVivo}})$ in an in vivo study for which scan and reconstruction parameters are constant. We normalise the in vitro qualification metric to values in the interval [0;1],

$$q_l^{\text{inVitro}} = \frac{1}{1+\exp(-\log(\sigma^{\text{inVitro}}((s_{tj})_{t\in\text{inVitro}})/\sigma^{\text{inVivo}}((s_{ij})_{i\in\text{inVivo}})}. \quad (3)$$

**InSilico Qualification: The associations of features and surrogates are Linear and Similar in vivo & in silico.** We use simulations of Gaussian, Poisson, Rayleigh and Gamma noise to generate associations of surrogates and features for each patient in silico. In one of these simulations, for example, the original image is synthetically manipulated to contain a higher amount of Gaussian noise. Furthermore, we applied other noise patterns induced by physical processes involved in CT, as we

expect these to be typically found within clinical practice: Poisson, Rayleigh and Gamma noise. Surrogates strongly associated with the analysed features in silico should in principle be used for calibration as they calibrate the respective features at least for simulated technical variation.

Unfortunately, simulations do not always realistically describe the impact of technical variation found in in vivo cohorts. Most simulations are likely oversimplifications of the real technical impact in in vivo data. Theoretically, a complex feature can show a completely different association regarding the impact of noise variation in vivo and in silico. On the other hand, inter-patient correlations of image information, as described in the inVivo qualification, are not neccessarily induced technically. For instance, a texture feature in a tumour may of course be biologically correlated with a surrogate within the adipose tissue in case of inflammation. An adjustment for the surrogate thus might reduce the impact of inflammation on the tumour as a side product, although this information might be diagnostically relevant. Thus, in vivo and in silico assessment used alone, have their methodological flaws. However, when both are sufficiently realistic, their description of the technical association between features $j$ and surrogate $l$ can be expected to be identical or at least similar.

The inSilico qualification criterion uses this concept by enforcing association of feature and surrogate in vivo, measured by the linear regression slope $\hat{\beta}^{\text{inVivo}}_{s,jl}$ of feature j w.r.t. surrogate $l$ over the cohort, and association in silico, measured by the cohort-average $\langle\hat{\beta}^{\text{inSilico}}_{s,jl}\rangle$ of the slope of feature $j$ w.r.t. surrogate $l$ over the simulated variations, to be similar. Biological associations between feature space and surrogate can thus be made improbable as synthetic simulations induce technical variations only. Similarity practically is a hint for the realism of the simulation itself, as the generated association between feature and surrogate in silico can be re-identified in actual in vivo patient data.

Since we screened for correlations between features and surrogates in vivo, it should be ensured that these associations are approximately linear, as the used correlation is a measure of linear association only. Therefore, the Pearson's correlation coefficient between feature $j$ and surrogate $l$ in silico is averaged over all observations within the cohort $\langle R^{\text{inSilico}}_{\text{Pearson}}((r_{ij})_{i\in P},(s_{il})_{i\in P})\rangle$ to guarantee a linear association in silico. The simulation itself can be assumed to be approximately correct for the feature as explained regarding the similarity of associations. An enforcement of linearity also acts as 'Occam's razor'-like regularisation, as the problem of finding a complex association of a feature with few surrogates is transferred to the task of identifying simple linear associations by the examination of a large number of surrogates.

We construct this qualification criterion as a multiplication of the similarity between slope and linearity. The inSilico qualification criterion for surrogate $l$ given a feature $j$ is normalised to values in the interval [0;1],

$$q^{\text{inSilico}}_{jl} = \max(\langle R^{\text{inSilico}}_{\text{Pearson}}((r_{ij})_{i\in P},(s_{il})_{i\in P})\rangle)\begin{cases} |\beta^{\text{inVivo}}_{s,jl}/\langle\hat{\beta}^{\text{inSilico}}_{s,jl}\rangle| & \text{if } \hat{\beta}^{\text{inVivo}}_{s,jl} < \langle\hat{\beta}^{\text{inSilico}}_{s.jl}\rangle \\ |\langle\hat{\beta}^{\text{inSilico}}_{s,jl}\rangle/\hat{\beta}^{\text{inVivo}}_{s,jl}| & \text{if } \hat{\beta}^{\text{inVivo}}_{s,jl} \geq \langle\hat{\beta}^{\text{inSilico}}_{s.jl}\rangle \end{cases}. \tag{4}$$

Of all simulations, the simulation with the largest value $q^{\text{inSilico}}_{jl}$ is used and the association between feature and surrogate is labelled accordingly as 'Poisson', 'Gaussian' etc.

**Statistical Qualification: Additivity of biological and technical association.** In order to properly calibrate a feature $j$ for technical variation, the technical variation has to be additive, i.e. orthogonal and statistically independent to the examined biological variation. It follows that no interactions must occur. Assuming interactions, the impact of technical variation on the feature could not be separated from the impact of the biological variation of interest. As the statistical qualification criterion depends on the concrete target variable and is thus disease-specific, different surrogate variables might be qualified, depending on the diagnostic question. Interaction existence is tested via decomposition of the feature in a biological variation, a technical variation and an interaction term of technical and biological variation term[5],

$$r_{ijl} = r_{0j} + \beta_{b,jl}b_i + \beta_{s,jl}s_{il} + \beta_{s\times b,jl}(s_{il}\times b_i), j\in R, l\in S. \tag{5}$$

A linear fit for this formula then yields estimates for the regression coefficients $\beta_{b,jl}$, $\beta_{s,jl}$ and $\beta_{s\times b,jl}$. The t-values are trivially calculated from the regression coefficients. The statistical qualification criterion uses the absolute t-value of the interaction term $|t_{b\times s,jl}|$ relative to the sum of absolute t-values of the biological $|t_{b,jl}|$ and technical variation $|t_{s,jl}|$. We normalise this criterion to values in the interval [0;1],

$$q^{\text{orthog}}_{jl} = 1 - \begin{cases} \frac{|t_{b\times s,jl}|}{|t_{b,jl}|+|t_{s,jl}|} & \text{if } |t_{b\times s,jl}| \leq |t_{b,jl}|+|t_{s,jl}| \\ 1 & \text{if } |t_{b\times s,jl}| > |t_{b,jl}|+|t_{s,jl}| \end{cases}. \tag{6}$$

## B. Pseudo Code

**Algorithm 1:** Surrogate Selection

**Input:** feature identifiers $R$, surrogate identifiers $S$, label identifier $b$, patient identifiers $P$, simulation identifiers $M$, weights $\Theta$, inVivo data $D_{\text{inVivo}}$, inVitro data $D_{\text{inVitro}}$, inSilico data $D_{\text{inSilico}}$
**Output:** set of qualified, selected surrogates $S^q_{r,\Theta}$ for the respective features $R$ and according to the weights $\Theta$
**Algorithm:** surrogateSelection($R, S, b, P, M, \Theta, D_{\text{inVivo}}, D_{\text{inSilico}}, D_{\text{inVitro}}$)

```
begin
    for j in R do
        for l in S do
            q_jl^inVivo, β_jl^inVivo ← inVivoQualification(j, l, D_inVivo)
            q_jl^inSilico ← inSilicoQualification(j, l, β_jl^inVivo, P, M, D_inSilico)
            q_l^inVitro ← inVitroQualification(l, D_inVitro, D_inVivo)
            q_jl^orthog ← statisticalQualification(j, l, b, D_inVivo)
            // calculate qualification according to the weights
            Q_jl(Θ) = θ^inVivo q_jl^inVivo + θ^inSilico q_jl^inSilico + θ^inVitro q_jl^inVitro + θ^orthog q_jl^orthog
        end
    end
    // select surrogates according to their qualification for the features R and the weights Θ
    Q_min = 1.0
    S^q_{R,Θ} ← {l ∈ S | Q_jl(Θ) > Q_min, j ∈ R}
    return S^q_{R,Θ}
end
```

**Algorithm 2:** Loss Functions for *Predictive* and *Stabilisation* Mode

**Input:** feature identifiers $R$, surrogate identifiers $S$, label identifier $b$, patient identifiers $P$, simulation identifiers $M$, weights $\Theta$, parameter $\alpha$, inVivo data $D_{\text{inVivo}}$, inVitro data $D_{\text{inVitro}}$, inSilico data $D_{\text{inSilico}}$
**Output:** predictive loss $L$
**Algorithm:** predictiveLossFunction($R, S, b, P, M, \Theta, \alpha, D_{\text{inVivo}}, D_{\text{inSilico}}, D_{\text{inVitro}}$)

**begin**
- // select surrogates according to the chosen weights and the features $R$
- $S^q_{R,\Theta} \leftarrow$ **surrogateSelection**$(R, S, b, P, M, \Theta, D_{\text{inVivo}}, D_{\text{inSilico}}, D_{\text{inVitro}})$
- // fit model to predict label b by features $R$ and qualified surrogates $S^q_{R,\Theta}$
- ClassificationModel $\leftarrow$ **classificationFit**$(R, S^q_{R,\Theta}, b, D_{\text{inVivo}})$
- // get performance metric such as ROC-AUC
- Perf $\leftarrow$ **performanceMetric**(ClassificationModel)
- // loss is the sum of training and calibration loss
- $L_{\text{calib}} \leftarrow \frac{-\alpha}{\sum_{\theta \in \Theta} \theta}$
- $L_{\text{train}} \leftarrow -\text{Perf}$
- $L \leftarrow L_{\text{train}} + L_{\text{calib}}$
- return $L$

**end**

**Input:** feature identifiers $R$, surrogate identifiers $S$, label identifier $b$, patient identifiers $P$, simulation identifiers $M$, weights $\Theta$, parameter $\alpha$, inVivo data $D_{\text{inVivo}}$, inVitro data $D_{\text{inVitro}}$, inSilico data $D_{\text{inSilico}}$
**Output:** stabilisation loss $L$
**Algorithm:** stabilisationLossFunction($R, S, b, P, M, \Theta, \alpha, D_{\text{inVivo}}, D_{\text{inSilico}}, D_{\text{inVitro}}$)

**begin**
- **for** *j in R* **do**
  - // select surrogates according to the chosen weights and feature $j$
  - $S^q_{j,\Theta} \leftarrow$ **surrogateSelection**$(j, S, b, P, M, \Theta, D_{\text{inVivo}}, D_{\text{inSilico}}, D_{\text{inVitro}})$
  - // fit model to predict deviation of feature from mean := $\Delta(j, D_{\text{inVivo}})$ by qualified surrogates' deviation from mean := $\Delta(S^q_{j,\Theta}, D_{\text{inVivo}})$
  - RegressionModel$_j \leftarrow$ **regressionFit**$(\Delta(j, D_{\text{inVivo}}), \Delta(S^q_{j,\Theta}, D_{\text{inVivo}}))$
  - // get variance of feature explained by selected surrogates
  - Perf$_j \leftarrow$ **varianceExplained**(RegressionModel$_j$)
- **end**
- // loss is the sum of training and calibration loss; training loss is averaged over all features
- $L_{\text{calib}} \leftarrow \frac{-\alpha}{\sum_{\theta \in \Theta} \theta}$
- $L_{\text{train}} \leftarrow -$**avg**$_j(\text{Perf}_j)$
- $L \leftarrow L_{\text{train}} + L_{\text{calib}}$
- return $L$

**end**

**Algorithm 3:** Qualification Criteria

**Input:** feature identifier $j$, surrogate identifier $l$, inVivo data $D_{\text{inVivo}}$
**Output:** inVivo qualification $q_{jl}^{\text{inVivo}}$ of surrogate $l$ with respect to feature $j$
**Algorithm:** inVivoQualification$(j,l,D_{\text{inVivo}})$

```
begin
    // qualify inVivo
    q_jl^inVivo ← computePearsonCorrelation(j, l, D_inVivo)
    // calculate inVivo slope
    β_jl^inVivo ← computeSlope(j, l, D_inVivo)
    return q_jl^inVivo, β_jl^inVivo
end
```

**Input:** feature identifier $j$, surrogate identifier $l$, patient identifiers $P$, simulation identifiers $M$, inVivo slope $\beta_{jl}^{\text{inVivo}}$, inSilico data $D_{\text{inSilico}}$
**Output:** inSilico qualification $q_{jl}^{\text{inSilico}}$ of surrogate $l$ with respect to feature $j$
**Algorithm:** inSilicoQualification$(j,l,\beta_{jl}^{\text{inVivo}},P,M,D_{\text{inSilico}})$

```
begin
    for m in M do
        for p in P do
            // compute inSilico correlation for feature r and surrogate s for patient p and simulation m
            R_Pearson^inSilico(j, l, m, p) ← computePearsonCorrelation(j, l, D_inSilico(m, p))
            // compute inSilico slope for feature r and surrogate s for patient p and simulation m
            β_jl^inSilico(m, p) ← computeSlope(j, l, D_inSilico(m, p))
        end
        // average inSilico slope and correlation over all patients
        β_jl^inSilico(m) ← avg_p(β_jl^inSilico(m, p))
        R_Pearson^inSilico(j, l, m) ← avg_p(R_Pearson^inSilico(j, l, m, p))
        // get score for simulation m
        if β_jl^inSilico(m) > β_jl^inVivo then
            q_jl^inSilico(m) ← R_Pearson^inSilico(j, l, m) · β_jl^inSilico(m) / β_jl^inVivo
        else
            q_jl^inSilico(m) ← R_Pearson^inSilico(j, l, m) · β_jl^inVivo / β_jl^inSilico(m)
        end
    end
    // use qualification of most suitable simulation
    q_jl^inSilico ← max_m(q_jl^inSilico(m))
    return q_jl^inSilico
end
```

**Input:** feature identifier $j$, surrogate identifier $l$, label identifier $b$, inVivo data $D_{\text{inVivo}}$
**Output:** statistical qualification $q_{jl}^{\text{orthog}}$ of surrogate $l$ with respect to feature $j$
**Algorithm:** statisticalQualification$(j,l,b,D_{\text{inVivo}})$

```
begin
    // decompose feature in surrogate, label and interaction effect and get t-values of s_l, b and interaction effect b × s_l
    t_{b,jl}, t_{s,jl}, t_{b×s,jl} ← linearFit(r_j ∼ s_l + b + (b × s_l), D_inSilico)
    if t_{b×s,jl} > t_{s,jl} + t_{b,jl} then
        q_jl^orthog ← 0
    else
        q_jl^orthog ← 1 − t_{b×s,jl} / (t_{b,jl} + t_{s,jl})
    end
    return q_jl^orthog
end
```

**Input:** surrogate identifier $l$, inVitro data $D_{\text{inVitro}}$, inVivo data $D_{\text{inVivo}}$
**Output:** inVitro qualification $q_{l}^{\text{inVitro}}$ of surrogate $l$
**Algorithm:** inVitroQualification$(l,D_{\text{inVitro}},D_{\text{inVivo}})$

```
begin
    σ_l^inVitro ← computeStdDev(l, D_inVitro)
    σ_l^inVivo ← computeStdDev(l, D_inVivo)
    q_l^inVitro ← 1 / (1 + exp(−log(σ_l^inVitro / σ_l^inVivo)))
    return q_l^inVitro
end
```

**Algorithm 4:** Technome - *Predictive* and *Stabilisation* Mode

**Input:** feature identifiers $R$, surrogate identifiers $S$, label identifier $b$, patient identifiers $P$, simulation identifiers $M$, parameter $\alpha$, inVivo data $D_{\text{inVivo}}$, inVitro data $D_{\text{inVitro}}$, inSilico data $D_{\text{inSilico}}$
**Output:** classifier $f^{\text{clf}}$ with implicit calibration for features $R$ and label $b$
**Algorithm:** technomePrediction$(R, S, b, P, M, D_{\text{inVivo}}, D_{\text{inSilico}}, D_{\text{inVitro}})$

**begin**
- $\Theta^* = \text{argmin}_\Theta(\textbf{predictiveLossFunction})$
- $S^q_{R,\Theta} \leftarrow \textbf{surrogateSelection}(R, S, b, P, M, \Theta^*, D_{\text{inVivo}}, D_{\text{inSilico}}, D_{\text{inVitro}})$
- // fit model to predict label b by feature identifiers $R$ and qualified surrogates $S^q_{R,\Theta}$
- $f^{\text{clf}} \leftarrow \textbf{classificationFit}(R, S^q_{R,\Theta}, b, D_{\text{inVivo}}))$
- return $f^{\text{clf}}$

**end**

**Input:** feature identifiers $R$, surrogate identifiers $S$, label identifier $b$, patient identifiers $P$, simulation identifiers $M$, parameter $\alpha$, inVivo data $D_{\text{inVivo}}$, inVitro data $D_{\text{inVitro}}$, inSilico data $D_{\text{inSilico}}$
**Output:** regression model $g^{\text{reg}}_j$ for explicit calibration of feature $j$
**Algorithm:** technomeStabilisation$(R, S, b, P, M, D_{\text{inVivo}}, D_{\text{inSilico}}, D_{\text{inVitro}})$

**begin**
- $\Theta^* = \text{argmin}_\Theta(\textbf{stabilisationLossFunction})$
- $S^q_{j,\Theta} \leftarrow \textbf{surrogateSelection}(j, S, b, P, M, \Theta^*, D_{\text{inVivo}}, D_{\text{inSilico}}, D_{\text{inVitro}})$
- // fit model to predict deviation of feature from mean := $\Delta(j, D_{\text{inVivo}})$ by qualified surrogates' deviation from mean := $\Delta(S^q_{j,\Theta}, D_{\text{inVivo}})$
- $g^{\text{reg}}_j \leftarrow \textbf{regressionFit}(\Delta(j, D_{\text{inVivo}}), \Delta(S^q_{j,\Theta}, D_{\text{inVivo}}))$
- return $g^{\text{reg}}_j$

**end**

**Algorithm 5:** Validation Functions

**Input:** feature identifiers $R$, surrogate identifiers $S$, label identifier $b$, patient identifiers $P$, simulation identifiers $M$, inVivo training data $D^{\text{train}}_{\text{inVivo}}$, inVivo test data $D^{\text{test}}_{\text{inVivo}}$, inSilico training data $D^{\text{train}}_{\text{inSilico}}$, inVitro data $D_{\text{inVitro}}$
**Output:** stabilisation performance on unseen test data
**Algorithm:** validateStabilisation$(R, S, b, P, M, D^{\text{train}}_{\text{inVivo}}, D^{\text{test}}_{\text{inVivo}}, D^{\text{train}}_{\text{inSilico}}, D_{\text{inVitro}})$

**begin**
- **for** *j in R* **do**
  - $g^{reg}_j \leftarrow \textbf{technomeStabilisation}(R, S, b, P, M, D^{\text{train}}_{\text{inVivo}}, D^{\text{train}}_{\text{inSilico}}, D_{\text{inVitro}})$
  - // use deviation of qualified surrogates' deviation from their mean (or another fixed value) on test data to calibrate feature $j$ for patient $i$
  - $r^*_{ij} \leftarrow r_{ij} - g^{\text{reg}}_j(\Delta(S^q_{j,\Theta}, D^{\text{test}}_{\text{inVivo}}))$
  - $\text{VarianceReduction}_j \leftarrow 1 - \frac{\sigma((r^*_{ij})_{i \in P}, D^{test}_{inVivo})}{\sigma((r_{ij})_{i \in P}, D^{\text{test}}_{\text{inVivo}})}$
- **end**
- $\text{Perf} \leftarrow \textbf{avg}_j(\text{VarianceReduction}_j)$
- return Perf

**end**

**Input:** feature identifiers $R$, surrogate identifiers $S$, label identifier $b$, patient identifier $P$, simulation identifiers $M$, inVivo training data $D^{\text{train}}_{\text{inVivo}}$, inVivo test data $D^{\text{test}}_{\text{inVivo}}$, inSilico training data $D^{\text{train}}_{\text{inSilico}}$, inVitro data $D_{\text{inVitro}}$
**Output:** predictive performance on unseen test data
**Algorithm:** validatePrediction$(R, S, b, P, M, D^{\text{train}}_{\text{inVivo}}, D^{\text{test}}_{\text{inVivo}}, D^{\text{train}}_{\text{inSilico}}, D_{\text{inVitro}})$

**begin**
- $f^{\text{clf}} \leftarrow \textbf{technomePrediction}(R, S, b, P, M, D^{\text{train}}_{\text{inVivo}}, D^{\text{train}}_{\text{inSilico}}, D_{\text{inVitro}})$
- // incorporate qualified surrogates $S^q_{R,\Theta}$ for features $R$ to predict unseen label $b$ of test data
- $\text{Perf} \leftarrow f^{\text{clf}}(R, S^q_{R,\Theta}, D^{\text{test}}_{\text{inVivo}})$
- return Perf

**end**

**Algorithm 6:** Main - 10-fold CV and SSD for COPD, mCRC and Phantom Data

```
// collect 10 random seeds for 10-fold CV or 5 for SSD
randomSeeds ← getRandomSeeds // evaluate
for k in randomSeeds do
    // For SSD, 0.9 is substituted by 0.2
    D^train_Phantom, D^test_Phantom ← randomSplit(D_Phantom, 0.9, k)
    D^train_COPD,inVivo, D^test_COPD,inVivo ← randomSplit(D_COPD,inVivo, 0.9, k)
    D^train_COPD,inSilico, D^test_COPD,inSilico ← randomSplit(D_COPD,inSilico, 0.9, k)
    D^train_mCRC,inVivo, D^test_mCRC,inVivo ← randomSplit(D_mCRC,inVivo, 0.9, k)
    D^train_mCRC,inSilico, D^test_mCRC,inSilico ← randomSplit(D_mCRC,inSilico, 0.9, k)


    // For phantom validation, only the inVivo qualification is used
    PerfPhantom_k ← validateStabilisation(R_Aerts, S, D^train_Phantom, D^test_Phantom, D_inVitro)
    // For COPD validation, all qualification criteria are use
    PerfCOPD_k ← validatePrediction(R_LAA, S, b_emphysema, P_COPD, M, D^train_COPD,inVivo, D^test_COPD,inVivo, D^train_COPD,inSilico, D^test_COPD,inSilico, D_inVitro)
    // For mCRC validation, all qualification criteria are used
    PerfmCRC_k ← validatePrediction(R_Aerts, S, b_1−ys, P_mCRC, M, D^train_mCRC,inVivo, D^test_mCRC,inVivo, D^train_mCRC,inSilico, D^test_mCRC,inSilico, D_inVitro)
end
PerfPhantom ← microAvg_k(PerfPhantom_k)
PerfCOPD ← microAvg_k(PerfCOPD_k)
PerfmCRC ← microAvg_k(PerfmCRC_k)
```

## C. Rationale for Qualification Criteria and Further Literature

We briefly summarise the status-quo to identify technical variation and reduce it in imaging studies. In general we find 3 standard approaches. We gain knowledge about feature stability and the possibility to calibrate features (i) from image manipulation procedures such as adding noise or denoising, (ii) from phantom scans or (iii) data-driven from real data.

The standard approach to examine the impact of $T^R$ on features is to extract the features in an **anthropomorphic phantom** and change the scan parameters[6–10]. The potential disadvantage of this method is that phantom measurements may not always realistically reproduce the non-reducible technical variation, that occurs when scanning biological objects with all their variation in size, shape and tissue characteristics. Additionally, the effect of scan parameters on different morphologies of the analyzed ROI is assumed to distinct.

Other straightforward approaches in the literature to reduce technical impact are **de-noising[11], image normalisation and artifact reduction filters**. De-noising especially reduces inter-patient noise variation. It smoothes out noise and can help to increase diagnostic power. The potential disadvantage is that no de-noising or in general image standardisation algorithm will completely preserve the measured structure. Thus the feature space, especially texture features, loses fine-grained information that could potentially be relevant for diagnosis or quantitative imaging biomarker identification. This can also be true for advanced concepts that standardise the images within the reconstruction process such as iterative reconstruction[12,13]. Also, appropriate standardisation approaches have to be carefully chosen according to the imaging scenario at hand, as seen for the white stripe normalisation[14] that adjusts the intensity distribution in MR images according to the intensity distribution in a reference region but shows lower reproducibility than covariate approaches. General approaches may tend to lose more biologically relevant information than specifically tailored ones.

In functional MRI (fMRI), **proportional scaling** is used to normalise features. A feature value is divided by the surrogate — in fMRI most often the global signal — in case of positive correlation with the feature and multiplied in case of negative correlation[15]. The disadvantage of proportional scaling is that when the impact of technical variation on a feature is low, a division or multiplication with a surrogate introduces a large error. A difference between two observations can then result solely from a difference in the surrogate while the feature of interest differs only slightly. The impact of technical variation on a feature is not known. An a-priori information to estimate the impact of technical variation on the feature in advance is missing.

**Digital phantoms** of physical structures with varying morphology are computed to simulate biological variation. For these structures, technical variation can also be simulated. The phantoms and simulations are used to discover the impact of biological and technical variation on the feature space in silico. It is also possible to assess feature stability by the simulation of technical variation for real patient data[16]. The potential disadvantage of digital phantoms is, that those can be coarse oversimplifications of real biological structures. Additionally, yet it remains an assumption that the used simulation of technical variation mimics the impact of real technical variation.

**Covariate approaches** based on the General Linear Model are used to adjust the measured feature values or intensity distributions for the impact of a surrogate, i.e., the values are linearly transformed by parameters derived from mutual covariance analyses between surrogate and ROI feature or intensity distribution. In some MRI publications the measurements are adjusted for a surrogate like the global signal intensity[15]. A special case is RAVEL[17], where a covariate vector is determined by a surrogate variable analysis[18] in a control region cerebrospinal fluid. This approach achieves superior performance compared to the white stripe normalisation. The disadvantage of this method is that it is not sure whether the association of a surrogate with a feature is induced biologically or technically. A technically-induced correlation is assumed and identfied as a limitation by the author. It is not known if e.g. a global signal in fMRS, if used as a surrogate, is biologically meaningful and not merely a technical variation. Additionally, it is not clear whether the impact of technical variation on a feature is indeed linear, which is assumed by these approaches.

It is also possible that features are inherently calibrated by the integration of a term, that stabilises them against the impact of technical variation, this is called **invariant feature engineering**. The feature thus becomes approximately invariant to technical variation. An example for this approach to calibrate muscle density measurements can be found in our previous work[19]. The stabilisation term is most often based on an adaptation to the mean or standard deviation of the intensity distribution of a CR. The disadvantage of this method is that the term is determined manually by trial-and-error. Up to date only relatively few and simple measurements are calibrated by this approach.

In conclusion, all status-quo techniques have potential obstacles, thus our aim was to develop a holistic approach. We integrate data-driven approaches based on internal calibration applied on in vivo data, in vitro phantom studies and in silico techniques, i.e. simulations. We do not want to use de-noising techniques or artifact reduction filters. For the inSilico qualification, the biological information is superimposed by simulation of technical variation, thus the biological information should be preserved. As it is known, that the noise and artifact expression is different in every region of an image, we also integrate CRs in local proximity to the ROI analysed.

In vitro feature analyses based on phantom measurements can potentially yield different results than in the in vivo case, thus we use further qualification criteria. The patterns to stabilise the features are learned on in vivo data. As invariant feature

engineering can be a tedious process, the qualification criteria aim to enable automated feature calibration by the analysis of in vivo patterns. Covariate approaches assume a linear technically induced association between covariates and features. The inSilico qualification criterion analyses, if the dependency is really induced technically, is linear and if the technical impact can be abstracted from the diagnostic relevant biological variation. Proportional scaling misses a-priori information, why it can introduce a large error for robust features. SALSA integrates two stability tests, thus robust features are not corrected as no surrogates are qualified. In comparison to simulations the technome compares the in silico patterns with patterns found in vivo, and can thus justify the use of simulations. In comparison to analyses with digital phantoms, the technome quantifies technical impact from in vivo data.

All approaches above are most often not tailored for a specific diagnosis or machine learning-based prediction. Therefore we introduce the technome *predictive* mode. The *predictive* mode could potentially enable a screening for new manifestations of in vivo image quality for specific biomarkers. This taking into account complex factors like body temperature and the impact of a contrast bolus on the radiome, which can solely be identified in vivo. The technome quantifies feature stability in silico by simulations and data-driven on in vivo data and those insights can be displayed to the user to gain insight on features used in a biomarker study.

The technome is dedicated to the radiome. Contrary to imaging physics and reconstruction approaches, the approach does not guarantee a consistent image quality as a basis for the qualitative-radiological diagnosis. Also it is not designed to standardise 3D images for the impact of technical variation. It is designed to calibrate the radiome with respect to the impact of technical variation. Our method is of lower dimensionality than image standardisation algorithmes because the amount of automatically extracted image features is usually lower than the amount of image voxels by several orders of magnitude. The dimensionality further decreases if only diagnostically features need to be calibrated.

## References


1. Seifert, S. *et al.* Hierarchical parsing and semantic navigation of full body ct data. In *Medical Imaging 2009: Image Processing*, vol. 7259, 725902 (International Society for Optics and Photonics, 2009).
2. Mühlberg, A., Kärgel, R., Wels, M. & Sühling, M. A method to determine at least one object feature of an object. *Eur. Pat. Appl. No. 17196657.5* (October 2017).
3. Fazlollahi, A. *et al.* A framework to objectively identify reference regions for normalizing quantitative imaging. In *International Conference on Medical Image Computing and Computer-Assisted Intervention*, 65–72 (Springer, 2018).
4. Lee, D. C., Hoffmann, P. F., Kopperdahl, D. L. & Keaveny, T. M. Phantomless calibration of ct scans for measurement of bmd and bone strength—inter-operator reanalysis precision. *Bone* **103**, 325–333 (2017).
5. Cox, D. R. Interaction. *Int. Stat. Rev. Int. de Stat.* 1–24 (1984).
6. Yasaka, K. *et al.* Precision of quantitative computed tomography texture analysis using image filtering: A phantom study for scanner variability. *Medicine* **96** (2017).
7. Shafiq-ul Hassan, M. *et al.* Intrinsic dependencies of ct radiomic features on voxel size and number of gray levels. *Med. physics* **44**, 1050–1062 (2017).
8. Larue, R. T. *et al.* 4dct imaging to assess radiomics feature stability: An investigation for thoracic cancers. *Radiother. Oncol.* **125**, 147–153 (2017).
9. Lu, L., Ehmke, R. C., Schwartz, L. H. & Zhao, B. Assessing agreement between radiomic features computed for multiple ct imaging settings. *PloS one* **11**, e0166550 (2016).
10. Kim, H. *et al.* Impact of reconstruction algorithms on ct radiomic features of pulmonary tumors: analysis of intra-and inter-reader variability and inter-reconstruction algorithm variability. *PloS one* **11**, e0164924 (2016).
11. Diwakar, M. & Kumar, M. A review on ct image noise and its denoising. *Biomed. Signal Process. Control.* **42**, 73–88 (2018).
12. Donoho, D. L. *et al.* Compressed sensing. *IEEE Transactions on information theory* **52**, 1289–1306 (2006).
13. Christianson, O. *et al.* An improved index of image quality for task-based performance of ct iterative reconstruction across three commercial implementations. *Radiology* **275**, 725–734 (2015).
14. Shinohara, R. T. *et al.* Statistical normalization techniques for magnetic resonance imaging. *NeuroImage: Clin.* **6**, 9–19 (2014).
15. Aguirre, G., Zarahn, E. & D'Esposito, M. The inferential impact of global signal covariates in functional neuroimaging analyses. *Neuroimage* **8**, 302–306 (1998).

**16.** Zwanenburg, A. *et al.* Assessing robustness of radiomic features by image perturbation. *Sci. reports* **9**, 614 (2019).

**17.** Fortin, J.-P. *et al.* Removing inter-subject technical variability in magnetic resonance imaging studies. *NeuroImage* **132**, 198–212 (2016).

**18.** Leek, J. T. & Storey, J. D. Capturing heterogeneity in gene expression studies by surrogate variable analysis. *PLoS genetics* **3**, e161 (2007).

**19.** Mühlberg, A., Museyko, O., Laredo, J.-D. & Engelke, K. A reproducible semi-automatic method to quantify the muscle-lipid distribution in clinical 3d ct images of the thigh. *PloS one* **12**, e0175174 (2017).
